# Integrated causal-predictive machine learning models for tropical cyclone epidemiology

**DOI:** 10.1093/biostatistics/kxab047

**Published:** 2021-12-28

**Authors:** Rachel C Nethery, Nina Katz-Christy, Marianthi-Anna Kioumourtzoglou, Robbie M Parks, Andrea Schumacher, G Brooke Anderson

**Affiliations:** Department of Biostatistics, Harvard T.H. Chan School of Public Health, 655 Huntington Ave, Boston, MA, USA; Department of Statistics, Harvard University, 1 Oxford St, Cambridge, MA, USA; Department of Environmental Health Sciences, Columbia Mailman School of Public Health, 722 W. 168th Street, New York City, NY, USA; Department of Environmental Health Sciences, Columbia Mailman School of Public Health, 722 W. 168th Street, New York City, NY, USA; Cooperative Institute for Research in the Atmosphere, Colorado State University, 3925A West Laporte Ave, Fort Collins, CO, USA; Department of Environmental & Radiological Health Sciences, Colorado State University, 122A Environmental Health Building, Fort Collins, CO, USA

**Keywords:** Causal inference, Environmental epidemiology, Extreme weather events, Hurricanes, Latent factor model, Matrix completion

## Abstract

Strategic preparedness reduces the adverse health impacts of hurricanes and tropical storms, referred to collectively as tropical cyclones (TCs), but its protective impact could be enhanced by a more comprehensive and rigorous characterization of TC epidemiology. To generate the insights and tools necessary for high-precision TC preparedness, we introduce a machine learning approach that standardizes estimation of historic TC health impacts, discovers common patterns and sources of heterogeneity in those health impacts, and enables identification of communities at highest health risk for future TCs. The model integrates (i) a causal inference component to quantify the immediate health impacts of recent historic TCs at high spatial resolution and (ii) a predictive component that captures how TC meteorological features and socioeconomic/demographic characteristics of impacted communities are associated with health impacts. We apply it to a rich data platform containing detailed historic TC exposure information and records of all-cause mortality and cardiovascular- and respiratory-related hospitalization among Medicare recipients. We report a high degree of heterogeneity in the acute health impacts of historic TCs, both within and across TCs, and, on average, substantial TC-attributable increases in respiratory hospitalizations. TC-sustained windspeeds are found to be the primary driver of mortality and respiratory risks.

## 1. Introduction

The US National Oceanic and Atmospheric Administration reports that tropical cyclones (TCs) impose the largest financial burden of any weather disasters in the United States, costing $945.9 billion since 1980 or roughly $21.5 billion per event (National Oceanic and Atmospheric Administration, 2020). TCs, which include hurricanes and tropical storms, often bring severe winds, rainfall, and flooding (Shultz *and others,* 2005), which can catalyze massive property and infrastructure damage. Due to the diverse types of hardships that can be set in motion by TC, the full spectrum of human health impacts of TCs are incompletely understood and unreliably quantified. Extreme weather events are known to cause both “direct” and “indirect” health impacts. It is well-appreciated that TCs introduce severe risks for accidental mortality and injuries (Centers for Disease Control and Prevention, 2005; Rappaport, 2014; Rappaport and Blanchard, 2016; Gong *and others,* 2007), such as drowning or blunt force trauma from falling debris, which are known as “direct” TC health impacts, that is, the TC can be clearly and conclusively identified as a causal mechanism. Direct TC health impacts are generally the focus of post-storm surveillance in the United States.

TCs can also “indirectly” elevate risk for a range of other adverse health events because, for example, they often cause power outages (Han *and others,* 2009; Klinger and Owen Landeg, 2014; Shultz and Galea, 2017), trigger mass evacuations (Lew and Wetli, 1996; Brunkard *and others,* 2008; Dosa *and others,* 2012), create psychological stress (Lutgendorf *and others,* 1995; Lenane *and others,* 2017), require clean-up (Centers for Disease Control and Prevention, 2004; Lew and Wetli, 1996), increase exposure to heat and pollution (Shultz and Galea, 2017), and interfere with normal medical care and medication use (Klinger and Owen Landeg, 2014; Gray and Hebert, 2007). Post-storm surveillance can hugely underestimate these indirect health impacts of TCs, as evidenced by Hurricane Maria. While surveillance initially attributed 64 deaths in Puerto Rico to the storm, later epidemiological studies estimated that the storm caused $>$2000 deaths (Kishore *and others,* 2018; Santos-Burgoa *and others,* 2018).

The literature on TC epidemiology has been dominated by single-storm studies (Lew and Wetli, 1996; Sharma *and others,* 2008; Kim *and others,* 2016; Kishore *and others,* 2018), seeking to quantify the total excess mortality or morbidity caused by a TC (including both direct and indirect effects). This focus on single storms is driven by the widely-recognized heterogeneity in TC health impacts. Recently, two large-scale studies estimated average health effects of all TC exposures in the United States spanning more than a decade (Yan *and others,* 2021; Parks *and others,* 2021). While these studies have revealed fundamental features of TC epidemiology, the results of single-storm studies may not generalize well, and multi-storm average health effects are too coarse to explain across-storm variability. Thus these studies have been unable deliver the targeted yet generalizable insights needed to guide strategic storm preparedness, which is believed to be one of the most effective tactics for minimizing TC health impacts (Thomalla and Schmuck, 2004; Shultz *and others,* 2005; Keim, 2008). A 2020 report by the National Academies of Sciences, Engineering, and Medicine stressed that in order to strengthen disaster resilience, improve responses, and quicken recoveries, the United States needs a uniform approach to quantifying disaster-related mortality and morbidity, as well as new analytical methods to enable estimation of disaster health impacts and the capacity to implement such methods on population-level data (National Academies of Sciences, Engineering, and Medicine, 2020).

The goal of our work is to inform strategic TC preparedness through development and application of a new modeling approach that (i) standardizes estimation of acute health impacts across past TCs, (ii) discovers common patterns and sources of heterogeneity in those health impacts, and (iii) enables identification of communities at highest health risk for future TCs. First, the proposed approach must incorporate a causal inference component that, when applied to historic data, estimates the excess adverse health events caused by past TCs (hereafter “health effects” or “health impacts”) at high spatial resolution in a standardized and transparent fashion. These estimates should capture both direct and indirect effects and should be adjusted for confounding. A TC’s health impact in a particular community may be influenced (i.e., modified) by a complex interplay among the features of the storm and the population (Keim, 2008), and understanding these drivers of heterogeneity is a key aim of our work. Thus, the second component of our approach is a predictive model relating the community- and TC-specific health impacts to the TC’s meteorological features and the socioeconomic/demographic features of the community. In addition to offering unprecedented insights into multi-storm TC epidemiology, this approach allows for community-specific prediction of the health impacts of an approaching TC with forecasted track and features. The predictive model could also be used to create general community-level TC health risk profiles based on a collection of representative future TC exposures. This tool represents a first step toward identifying communities at highest risk for adverse TC health impacts so that they can be targeted for immediate TC strategic preparedness and/or long-term efforts to increase resilience.

Building on a rich data set of recent historic US TC exposures and Medicare claims, we introduce an innovative statistical modeling approach that incorporates both the causal inference and predictive components described above. Our Bayesian machine learning method jointly fits causal inference sub-models to estimate the county-specific health effects of each historic TC, then passes these effect estimates into a predictive sub-model that captures relationships between county and TC features and health impacts. Leveraging recent advances in causal inference with observational pre/post treatment data, the causal sub-models employ a matrix completion approach that adjusts for unmeasured, time-varying confounding under mild assumptions (Athey *and others,* 2021). By joining the causal and predictive models in a Bayesian framework, we account for the uncertainty from all components, and predictions made using this model are accompanied by accurate uncertainty estimates, which are critical to assess their utility. This method can be widely used for characterizing and predicting the health impacts of extreme weather and climate events.

## 2. Methods

### 2.1. Data

The data are described briefly here, detailed descriptions are provided in Section S.1 of the Supplementary material available at *Biostatistics* online. All mortalities in the Medicare population and all respiratory disease hospitalizations, chronic obstructive pulmonary disease (COPD) hospitalizations, and cardiovascular disease (CVD) hospitalizations in the Medicare fee-for-service population are obtained for the period 1999–2015. For our analyses, these are aggregated to create daily county-level counts of each outcome. To characterize TC exposures, we leverage an open source data platform containing temporally detailed track and feature data for each Atlantic-basin TC during the period 1999–2015 that came within 250km of at least one eastern US county. More detail about this data platform, which is made available through the *hurricaneexposuredata* R package, is provided in Section S.1.2 of the Supplementary material and in Anderson *and others* (2020). For the causal inference component of our model, we classify each eastern US county as exposed or unexposed to each TC (equivalently treated/control for consistency with the causal inference literature). Following Yan *and others* (2021), counties that experience TC maximum sustained windspeeds of gale force or higher ($\geq 17.4$ m/s) at the population mean center are considered treated for a given TC and all others are controls.

After designating each county as treated or control for a given TC, we select a set of analytic treated and control counties for the TC that will be used to estimate its health impacts. To avoid instability in the analyses, any county, treated or control, is excluded from the analytic set for a given TC if it has Medicare fee-for-service population size less than 100 or if it experiences 5 or fewer of any of the health events under study during the TC study period (defined below). Further, among the control counties, we select as analytic controls only those that fall within 150 miles of at least one treated county (distance computed between county centroids). Exclusion of controls far from any treated county reduces noise, because vastly differing climate patterns can lead to seasonal trends in health outcomes that are not comparable across regions. Hereafter, when we reference the treated/control counties for a given TC, we are referring to the analytic treated/controls as defined here. We exclude TCs from the analysis entirely if they have no qualifying treated counties, if the total number of treated and control counties is less than 20, or if the number of control counties is less than 5.

**Fig. 1.**
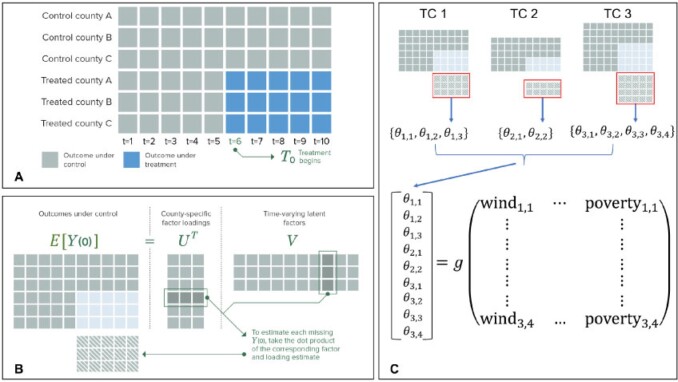
Example of panel data structure (A), illustration of matrix completion (B), and visual explanation of our integrated causal and predictive modeling approach (C).

For each TC and each health outcome, we create a matrix of panel data (Figure 1A), and these are central to the causal inference component of our model (Athey *and others,* 2021). The panel data matrix is composed of rows corresponding to each county in the analytic set for the given TC. Each row contains a time series of counts of the outcome in that county during the pre-specified TC study period. The TC study period is composed of a substantial time span prior to the storm, used to establish baseline trends in health outcomes in treated and control counties, as well as the period during and immediately after the storm, to estimate acute storm effects. For each TC, we define its study period as beginning 129 days prior to the TC’s first US approach and ending 11 days after the first approach. The TC’s first approach is the earliest date that the TC makes its closest approach to an exposed US county/counties. In practice, when constructing the panel data matrix, each county’s daily counts during the TC study period are aggregated into two-week cumulative counts, resulting in 10 2-week intervals. This temporal aggregation is performed because narrower time intervals lead to small counts and instability in the time series for many counties. We choose to include approximately 4.5 months of pre-TC baseline data because (i) this provides enough time points to reveal relationships in baseline trends in the health outcomes in treated vs. control counties while (ii) the time series covers a limited enough period that relationships between the time trends in treated and control counties would be expected to remain stable. The use of longer time series may introduce noise by capturing irrelevant long-term changes in relationships in baseline health across counties.

While Figure 1A is intentionally left general, in our analysis each panel data matrix has 10 columns, corresponding to the 10 2-week intervals in the TC study period, and the number of rows is determined by the number of treated and control counties. For each county, we classify each 2-week interval in the panel data matrix as a control or treatment period. For control counties, all 10 intervals are control periods. For treated counties, we consider the final 2-week period, beginning 2 days before and ending 11 days after the TC’s first approach, to be the treatment period, during which we seek to estimate the TC’s health impacts.

For the predictive component of our model, we also obtain detailed county-specific TC meteorological features (e.g., maximum sustained windspeed, duration of sustained wind speeds above 20 m/s) and socioeconomic and demographic characteristics for each TC and each exposed county. These are described in detail in Section S.1.3 of the Supplementary material.

### 2.2. Approach

For each health outcome, we construct a model composed of (i) causal inference sub-models for each TC to estimate the excess health events attributable to it in each impacted county and (ii) a predictive sub-model that relates these health effects to the TC and county features. We emphasize that each health outcome is modeled separately, with no transfer of information between the outcome-specific models. For the remainder of the section, we focus on the model for a single outcome. To emphasize the broader applicability of our approach, we present the methods using general notation, making connections back to our TC data structures for clarity.

#### 2.2.1. Causal inference sub-models

In this section, we describe the models that will be used to estimate the excess health events attributable to historic TCs. These models are applied separately to the data for each TC, which is part of a larger modularized model fitting scheme described in Section 2.2.2.

We denote the number of TCs in the study by $S$. In the causal inference sub-models, all data and parameters are storm-specific and should be indexed by an $s\in \left\lbrace 1, \dots, S \right\rbrace$. However, for clarity of presentation, we suppress these indices and introduce the causal inference concepts in the context of a single arbitrary TC. Let $i=1,...,N$ index the set of treated and control counties and $t=1,...,T$ index time intervals, so that the panel data matrix (Figure 1A) has dimensions $N\times T$, with counties in the rows and times in the columns.

For a given TC, we assume that all counties are untreated at $t=1$, and that once treatment begins for treated counties, they remain treated through $t=T$. We let $W$ denote the set of indices of treated counties. Let $D_{it}$ be a binary indicator of treatment of county $i$ by the TC at time $t$. Let $T_0$ denote the common time period when treatment is initiated in treated counties, such that all counties are untreated prior to $T_0$, and treatment occurs for treated counties at times $T_0$ through $T$ (see Figure 1A). While staggered treatment initiation times can be accommodated in this framework, we focus on a common treatment initiation time for clarity. Thus, $D_{it}=1$ if $\left\lbrace (i,t): i\in W, t \geq T_0\right\rbrace$, $D_{it}=0$ otherwise. Collectively, the set of all $t\in \left[ T_0, T\right]$ is referred to as the treatment period.

$Y_{it}$ is the observed number of health events for county $i$ at time $t$, and the panel data matrix of the outcomes is denoted $\mathbf{Y}$. Following Athey *and others* (2021), we formalize our causal inference approach using Rubin’s potential outcomes framework (Rubin, 1974), invoking assumptions given in Section S.3 of the Supplementary material. In short, in treated counties during treatment, we observe the outcome that occurs under treatment and we wish to compare it to an estimate of the “counterfactual” outcome that would have occurred in the absence of treatment. Formally, let $Y_{it} (0)$ be the potential outcome in county $i$ at time $t$ under control. For control counties at all times and for treated counties prior to the treatment period, $Y_{it} (0)=Y_{it}$. For treated counties during the treatment period, we instead observe the potential outcome under treatment, $Y_{it} (1)=Y_{it}$. The aim of the causal inference sub-model for each TC is to estimate the individual excess events (IEE), defined as $\theta_{i}=\sum_{t\geq T_0} \left[ Y_{it} (1)-Y_{it} (0) \right]$ for $i\in W$. Here, the word individual refers to individual units of analysis, in our case counties. Because $Y_{it}(1)=Y_{it}$ for $\left\lbrace (i,t): i\in W, t \geq T_0\right\rbrace$, our aim is to estimate the counterfactual outcome, $Y_{it} (0)$.

Both spatial and temporal confounding are possible in studies of the health impacts of TCs. For example, coastal TC-prone counties may have wealthier populations and wealth is associated with health. TCs are also more likely to occur during certain seasons and in certain climate conditions, which may independently affect health outcomes. With most observational study designs, causal inference analyses rely on the assumption of ignorable treatment assignment conditional on observed confounders (no unmeasured confounding). To address potential unmeasured confounding, we conceptualize each TC as a quasi-experiment, that is, a study design with nonrandomized treatment assignment but with pre- and post-treatment data available. In environmental health studies, quasi-experimental designs are the gold standard for assessing causality because certain types of unmeasured confounders can be controlled for by design (Dominici and Zigler, 2017).

Classic methods such as difference-in-differences allow for control for time-invariant unmeasured confounders. Recent machine learning approaches such as matrix completion (Athey *and others,* 2021) go further by allowing control for certain types of time-varying unmeasured confounders. This ability to adjust for time-varying unmeasured confounding is particularly critical in our TC application. Many potential confounders of TC health effects demonstrate complex seasonal patterns, for example, employment (Krane and Wascher, 1999), use of homeless shelters (Colburn, 2017), and infectious disease proliferation, but measurements of these variables are unavailable at the space-time resolution needed.

To estimate the health impacts of a TC in each treated county, we propose an adaptation of the matrix completion (MC) approach for conducting causal inference with quasi-experiments using panel data (Athey *and others,* 2021; Tanaka, 2021; Pang *and others,* 2021). MC is a machine learning technique for imputing missing values in a matrix, learning from patterns in observed entries in both the rows and columns. In our setting, the matrix with missing entries is the matrix of $Y_{it} (0)$ values, denoted $\mathbf{Y(0)}$. $\mathbf{Y(0)}$ is structured just like the panel data matrix, with missing entries in positions corresponding to the treated counties during the treatment period (Figure 1A, blue elements missing). Using a low-rank latent factor representation, MC learns from space-time trends in the non-missing data, that is, the outcomes for (1) control counties at all time periods and (2) treated counties prior to treatment, to impute the missing $Y_{it} (0)$. In this approach, the observed $Y_{it}(1)$ are treated as fixed and known and are omitted from the MC model. In settings with normally distributed data, MC can be framed as a low-rank factorization of $\mathbf{Y(0)}$ (or of its expectation), as illustrated in Figure 1B.

Because our outcomes are counts, we generalize the MC approach for causal inference to allow for count data likelihoods. MC models for count data were developed in other contexts (Gopalan *and others,* 2014), but do not follow epidemiologic conventions for modeling count data. We instead propose the following MC model for count data using a log link:

(2.1) \begin{equation*}\label{eq:bmc} \text{log}(E\left[Y_{it} (0)\right])=\alpha+\gamma_i+\psi_t+\mathbf{U}_i^T \mathbf{V}_t+\text{log}(p_{it}) \end{equation*}

$\alpha$ is a global intercept, $\gamma_i$ are county-specific deviations from the global intercept, and $\psi_t$ are time-specific deviations. $\mathbf{V}_t$ is a $K$-length ($K<<\min(N,T)$, unknown) vector of unobserved factors influencing the $Y_{it} (0)$ that vary over time but are common to all counties and $\mathbf{U}_i$ is a $K$-length vector of the unobserved county-specific effects of the $\mathbf{V}_t$ on $Y_{it} (0)$. Together, the $\mathbf{V}_t$ and $\mathbf{U}_i$ provide a low-dimensional representation of the space-time trends in the $Y_{it} (0)$ (see Figure 1B for an illustration in the case of normally distributed outcomes). $p_{it}$ is a scalar population size offset, to allow for rate outcomes. We prespecify $K$ based on exploratory principal component analyses. Although predictions from the model in (2.1) are identified, the individual parameters on the right hand side are not identifiable without further constraints. However, even if constrained to enable identifiability, these parameters are typically slow to converge and often provide little substantive insight because the estimated latent factors are difficult to interpret. Moreover, commonly used identifiability constraints (e.g., upper-triangularity of the matrix $\left[ \mathbf{U}_1,...,\mathbf{U}_N \right]'$) can compromise the model’s predictive ability. Thus, we recommend leaving the parameters unidentifiable and regarding this as a black-box model for predicting counterfactual outcomes—such approaches are widely used and well-validated (Dorie *and others,* 2019).

We fit the MC models using a negative binomial likelihood and uninformative prior distributions, collecting MCMC samples using the $\texttt{rstan}$ software package (Stan Development Team, 2020). Explicit modeling details are given in Section S.2 of the Supplementary material. For a treated county $i$ at post-treatment time $t$, we use the above model to collect $M$ MCMC samples from the posterior predictive distributions of the missing counterfactuals, denoted $Y_{it}^{(m)}(0)$ for $m=\left\lbrace 1,...,M\right\rbrace$, and use those to construct $M$ posterior samples of the IEE, as $\theta_i^{(m)}=\sum_{t\geq T_0} \left\lbrace Y_{it}-Y_{it}^{(m)} (0) \right\rbrace$ for $i\in W$.

The formal causal identifying assumptions for this model, originally specified by Pang *and others* (2021) are provided in Section S.3 of the Supplementary material. Under these assumptions, the $\mathbf{U}_i^T \mathbf{V}_t$ should capture all space-time trends in the $Y_{it}(0)$, including trends induced by time-varying confounders. Thus the resulting IEE can be identified, assuming trends in confounders do not change differentially in treated units (relative to controls) post-treatment.

In practice, both the excess number of events and the excess rate of events (per unit population) are of interest for understanding the epidemiology of extreme weather events. Thus we define the individual excess rate as $\theta^*_{i}=100\, 000\times (\theta_{i}/p_{iT})$. We also define TC-specific excess events as the cumulative excess events across all counties impacted by a TC, and TC-specific excess rate as the excess rate across all impacted counties. To compare with existing literature and evaluate overall health burdens, we also wish to summarize the estimated health effects across our entire study. To this end, we define the total excess events (TEE) for the full study to be the cumulative TC-attributable excess events summed over all TCs and counties, and the average excess rate (AER) to be the average of the excess rates across all county-level TC exposures in the study. Formal definitions of each estimand are given in Table S.3 of the Supplementary material. Posterior samples of these quantities can be constructed through simple transformations of the $\theta_i^{(m)}$.

#### 2.2.2. Bayesian modularization

In a classic Bayesian framework, a full likelihood is specified for the data, and the model components are fit jointly, permitting unrestricted information flow. However, in many real-world contexts, there is a need to propagate uncertainty between model components without allowing information to flow bi-directionally between all model components. This may be due to philosophical considerations, as in the case of Bayesian propensity score methods (McCandless *and others,* 2010; Zigler *and others,* 2013), or practical considerations, as complex models fit jointly may require prohibitive computation times or suffer from poor mixing. Of particular relevance, in complex model settings, one “suspect” model component may contaminate and adversely impact all other model components when sampling from the full posterior (Liu *and others,* 2009). These concerns have given rise to a literature on Bayesian modularization, in which information flows between certain sub-models weakly or not at all (Liu *and others,* 2009; Lunn *and others,* 2009; Plummer, 2015; Jacob *and others,* 2017). This is typically achieved by simplifying or ignoring some components of the posterior distribution.

We modularize our models in a manner that prevents information flow between the causal inference sub-models for each TC (as described above) yet allows information to flow uni-directionally from the causal models into the predictive model. This enables uncertainty in the TC health effect estimates to be accounted for when fitting the predictive model but does not allow the predictive model to inform the causal effect estimates. Explicit details are provided in Section S.4 of the Supplementary material. This modularization approach is motivated by both philosophy and computational feasibility. Primarily, we wish to prevent information from the predictive model from influencing the causal models. In our context, the predictive model is regarded as a “suspect” model component, because we do not anticipate that it corresponds to any “true” data generating mechanism, we only contend that it may provide insight into associations between TC health impacts and observed TC/county features, which may aid in identifying future high-risk communities. Because we are doubtful that the predictive model represents any true model, whereas we are confident in the ability of the causal models to accurately estimate counterfactuals under mild assumptions, we prefer to modularize in order to avoid contaminating the results of the causal models with information from the predictive model. Moreover, because the causal and predictive models imply incompatible data generating and confounding mechanisms for the $Y_{it}(0)$ (see Section S.4 of the Supplementary material), allowing the predictive model to inform the causal model would obscure the identifying assumptions needed to obtain causal effect estimates.

The absence of shared parameters or hierarchical structures across causal models (alongside the identifying assumptions in Section S.3 of the Supplementary material) prevents information sharing and de facto modularizes the causal models across TCs. Because our causal modeling approach is computationally intensive and involves many unidentifiable parameters, modularization across TCs is practical, as it improves mixing and reduces computation time by enabling parallel model-fitting. Moreover, because all parameters in the causal models are intended to capture unmeasured confounding structures that may be unique to each TC, sharing or shrinking these parameters across models may reduce model flexibility and compromise confounding adjustment (Hahn *and others,* 2018).

#### 2.2.3. Predictive sub-model

We develop a predictive model for each health outcome that captures the relationship between the county-specific TC health effects and the features of the TC and county (i.e., characterizing how such features modify TC health impacts). For clarity in this section, we reintroduce the storm-specific indices, but continue to focus on a single outcome-specific model. We let $\theta_{si}^{*(m)}$ be the individual excess rate posterior sample $\theta_i^{*(m)}$ for TC $s$, and $W_{s}$ be the set of indices of treated counties for TC $s$. Then, for a single fixed posterior draw $m$, we collect a posterior sample of the parameters $\beta$ from the (outcome-specific) predictive model:

\begin{equation*} \{\theta_{si}^{*(m)}=g(\mathbf{X}_{si};\mathbf{\beta}) \; | \; s \in \{1, \dots S\}, i \in W_{s} \},\end{equation*}

where $\mathbf{X}_{si}$ is a vector of predictors, that is, modifiers, of the county-specific TC effects, and $g()$ is an unspecified function parameterized by a vector of global parameters $\mathbf{\beta}$. In practice, $g()$ could take the form of any Bayesian predictive model. We recommend selecting $g$() based on cross-validation performance. We repeat this sampling with each $\theta_{si}^{*(m)}$ to obtain posterior samples $\left\lbrace \beta^{(1)},...,\beta^{(M)}\right\rbrace$ (Figure 1C).

#### 2.2.4. Prediction for future TCs

Using the posterior samples $\mathbf{\beta}^{(m)}$, we can draw corresponding posterior predictive samples of the health effect, $\theta_{\rm new}^{*(m)}$, for any set of predictor values $\mathbf{X}_{\rm new}$. To use the model for county-level prediction of the health impacts of a specific approaching TC, $\mathbf{X}_{\rm new}$ could be defined as the forecasted meteorological characteristics of the TC and socioeconomic and demographic characteristics of each county on its expected path. The predicted health impacts and uncertainties for each county can be used to identify counties at highest health risk. Alternatively, to create a long-term TC health risk profile for a county, many different $\mathbf{X}_{\rm new}$ vectors could be created using the meteorological characteristics of a collection of hypothetical, representative TC exposures, as well as the socioeconomic and demographic characteristics of the county. The resulting set of predictions can be summarized to give insight into future TC health risks the community may face, in both expected and extreme TC scenarios.

## 3. Results

### 3.1. Causal analysis

Fifty-three TCs and 2135 corresponding county-level TC exposures occurring during the period 1999–2015 are included in our analysis. In Table S.4 of the Supplementary material, we provide the name and year of each TC included in our study, the number of treated and control counties used in its causal model, and the rate of each health outcome among the treated and controls during the TC study period. Figure S.8 of the Supplementary material maps the number of TC exposures by county. Coastal counties in the Carolinas and the Gulf Coast region are repeatedly exposed, with some receiving as many as 15 TC exposures during our 17-year study period. For a discussion of the possible impacts of TC-related population displacement on our analyses, see Section S.5 of the Supplementary material.

We apply the MC models for each TC and health outcome with $K=4$ factors. $K=4$ was chosen because exploratory principal component analyses revealed that 4 factors explained around 70% of the variance in the $\mathbf{Y(0)}$ matrices (Section S.6 of the Supplementary material). This selection allows for preservation of critical variance without overfitting. We run the causal models using two MCMC chains, collecting 1000 post-burn-in samples from each. Traceplots of the $Y_{it}^{(m)}(0)$ indicated convergence.

**Fig. 2.**
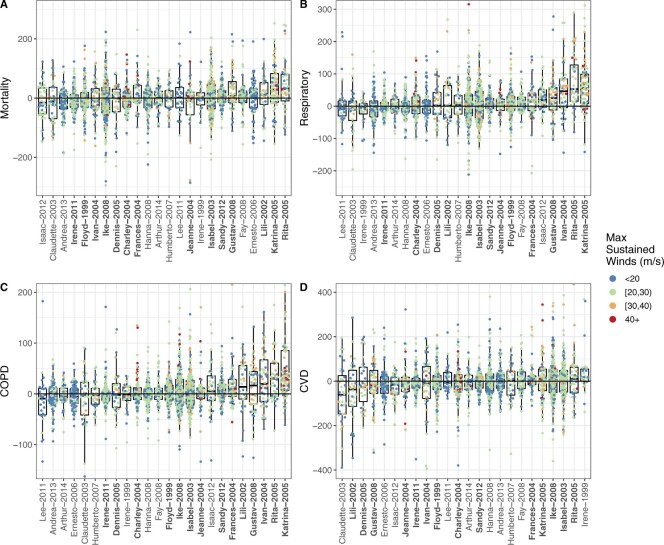
County-level excess rate estimates for mortality (A), respiratory hospitalizations (B), COPD hospitalizations (C), and CVD hospitalizations (D) for TCs that impacted $>$25 counties. The county excess rate is the estimated rate of excess events (per 100 000) in the county due to the TC. Distant outliers are cropped out for readability. Bolded TC labels indicate storm names that were subsequently retired—retirement occurs when a TC is so destructive that reusing the name is considered to be insensitive (National Hurricane Center, 2020).

**Fig. 3.**
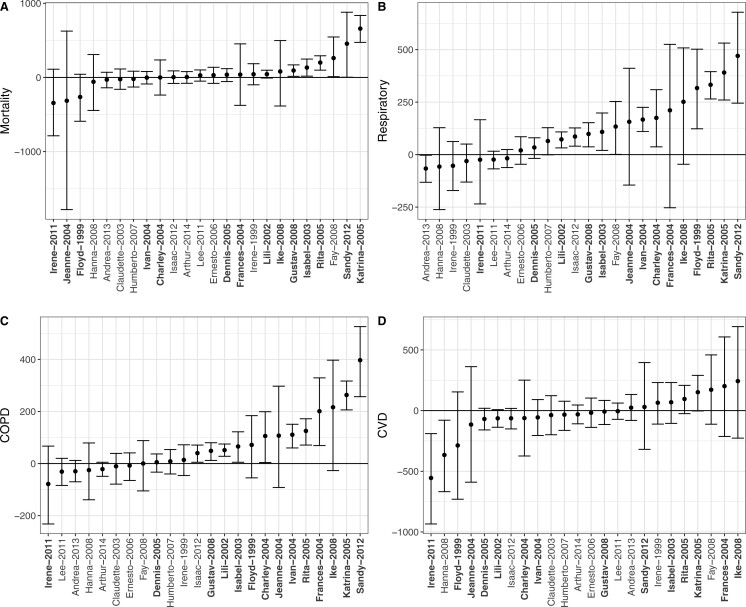
TC-specific excess events estimates and 95% predictive intervals for mortality (A), respiratory hospitalizations (B), COPD hospitalizations (C), and CVD hospitalizations (D) for TCs that impacted $>$25 counties. The TC-specific excess events is the sum of excess event estimates across all counties impacted by the TC. Bolded TC labels indicate storm names that were subsequently retired—retirement occurs when a TC is so destructive that re-using the name is considered to be insensitive (National Hurricane Center, 2020).

Recall that our analysis defines the treatment period as only the final 2-week time interval (beginning 2 days prior to the storm’s first approach and ending 11 days after). Thus, the IEE for county $i$ exposed to TC $s$, can be expressed simply as $\theta_{si}= Y_{iT} (1)-Y_{iT} (0)$, that is, the excess health events attributable to the TC at time $T$. For each of the four health outcomes, we have generated posterior samples of the IEE for each county impacted by each TC. We use these to construct posterior samples of the excess rates and the summary quantities described in Section 2.2.1. Hereafter, we refer to the posterior means for each parameter as the “estimates” from our models. Figure 2 and Figure S.9 of the Supplementary material show the county-specific and TC-specific excess rate estimates, respectively, for all TCs that impacted $>25$ counties. Figure S.10 of the Supplementary material and Figure 3 display the county-specific and TC-specific excess event estimates, respectively. These results illustrate the heterogeneity in TC health effects across counties and across storms.

We find that, on average, a county’s mortality rate increases slightly, though not significantly, during the 2-week treatment period, compared to the mortality rate expected in the absence of TC (AER: 2.58, 95% CI [$-$1.69 to 6.56]; TEE: 1228.86, 95% CI [$-$608.20 to 2731.07]). TC exposures cause larger and significant increases, on average, in respiratory hospitalizations (AER: 8.58 [4.34 to 11.86]; TEE: 2926.18 [1808.97 to 3940.02]) and COPD hospitalizations (AER: 4.57 [2.13 to 6.79]; TEE: 1532.80 [969.95 to 2106.10]). For each of these outcomes, we note that Hurricanes Katrina and Rita, which impacted largely overlapping sets of counties in the same year (2005), produced some of the largest adverse impacts (on both the excess event and the rate scale). We find that Hurricane Sandy caused huge increases in these outcomes specifically on the excess events scale, which is likely attributable to its impacts on the densely populated New York City area. Moreover, for each of these outcomes, Figure 2 and Figure S.10 of the Supplementary material suggest that counties experiencing higher TC windspeeds may be at increased risk.

For CVD, we find that hospitalization rates on average decrease during the 2-week period surrounding TC exposure (AER: $-$5.01 [$-$9.87 to $-$0.30]; TEE: $-$977.99 [$-$2246.53 to 222.10]). Previous studies have found decreases in CVD hospitalizations on the day of the storm but increases 2–3 days later (Yan *and others,* 2021; Parks *and others,* 2021). This finding is likely attributable in part to the fact that our hospitalization metric captures all inpatient hospitalizations, including planned procedures. The danger associated with venturing out during or immediately after a TC may motivate people to cancel planned procedures or treatment for chronic disease, so, even if emergency CVD hospitalizations increased during the TC exposure period, this may be outweighed by a decrease in non-emergency CVD hospitalizations.

### 3.2. Predictive analysis

The full set of candidate predictors is given in Section S.1.3 of the Supplementary material. We conduct predictive model selection using cross-validation as described in Section S.7 of the Supplementary material. The selected predictive model (common across health outcomes) is a Bayesian linear model with a spline on windspeed and year, with the remaining TC-related and socioeconomic/demographic predictors included as linear terms. For interpretability, we also provide results from a Bayesian linear regression model without the windspeed spline.

We fit the modularized Bayesian models and obtain 1000 post-burn-in samples of the predictive model parameters. Tables S.5 and S.6 of the Supplementary material give the posterior means and 95% credible intervals for the predictive model coefficients. To illustrate the importance of propagating uncertainties from the causal to predictive modules of our model, we also overlay our predictive model estimates and 95% CIs with those obtained by implementing the causal and predictive models separately without propagating uncertainty (Figure S.11 of the Supplementary material), that is, the causal models are fit to obtain posterior means of the causal effects, and the predictive model is fit to those posterior means.

**Fig. 4.**
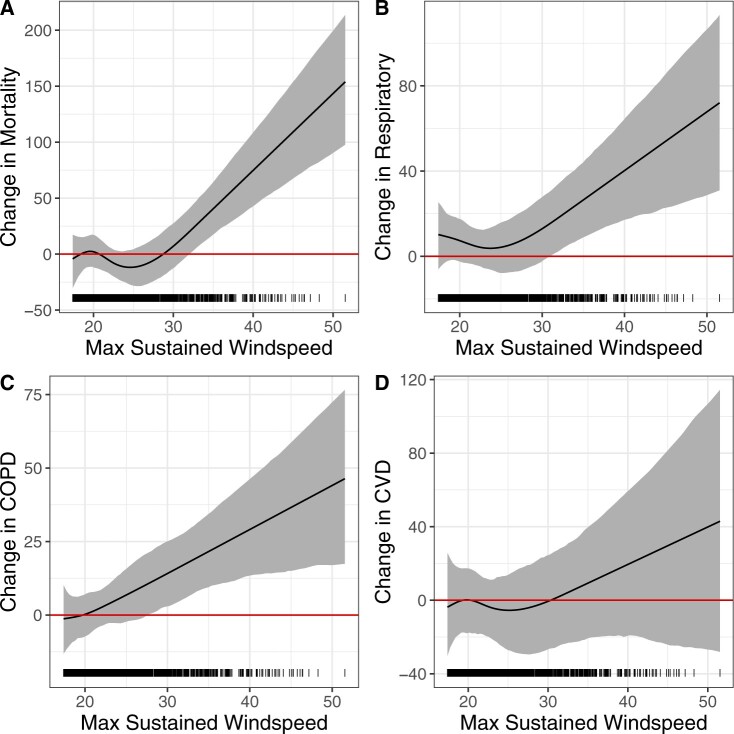
Relationship between maximum sustained windspeed and county-level excess rates per 100 000 of mortality (A), respiratory hospitalizations (B), COPD hospitalizations (C), and CVD hospitalizations (D).

In Figure 4, we show the windspeed splines and 95% credible intervals for each outcome. For each outcome, Table S.6 of the Supplementary material indicates that maximum sustained windspeed has the strongest association with health impacts, among the predictors considered. The splines illustrate that, as windspeeds increase beyond 30 m/s, we observe a sharp increase in TC-attributable mortality and respiratory and COPD hospitalizations. While we generally find a similar trend for windspeed and CVD hospitalizations, the relationship is weaker and more variable.

We also find that TC-attributable respiratory hospitalizations are positively associated with the duration of sustained windspeeds above 20 m/s (Table S.5 of the Supplementary material). For respiratory and COPD hospitalizations, we observe a negative association with total number of TC exposures during the study period, a proxy for TC exposure propensity (Table S.5 of the Supplementary material). This suggests that communities that are frequently hit may adapt in ways that decrease respiratory health impacts (e.g., bury power lines to decrease power outages, thereby decreasing risk for those dependent on electric-powered respiratory devices). Although few strong associations are detected for the county socioeconomic and demographic features, we find that predominately white communities tend to experience fewer TC-attributable COPD hospitalizations (Table S.5 of the Supplementary material).

### 3.3. Sensitivity analyses

We conduct a range of sensitivity analyses for both the causal and predictive components of our model (Section S.8 of the Supplementary material). For the causal models, we evaluate sensitivity to our definition of TC exposure and to specification of $K$. Because we have cumulative TC precipitation data only for years prior to 2012, we fit predictive models restricted to years 2011 and earlier and include a restricted cubic spline on cumulative precipitation as a predictor. In short, we find that our causal models are robust to these specifications and that precipitation is weakly, if at all, associated with the acute health impacts of TCs after adjusting for other factors.

## 4. Discussion

We have proposed and implemented an integrated causal and predictive modeling framework for systematically characterizing and predicting the health impacts of TCs in the United States, in order to inform pre-storm strategic preparedness efforts. This work offers several contributions to the existing literature on TC epidemiology. First, we have used a standardized causal inference approach to estimate county-level TC-attributable excess mortality and excess respiratory, COPD, and CVD hospitalizations (with uncertainties) in the Medicare population for nearly all Atlantic-basin TCs 1999–2015. These excess event estimates provide a more complete picture of TC health burdens than post-storm surveillance efforts or single-storm studies. We have also found that, controlling for a number of demographic and meteorological predictors, the maximum sustained windspeed experienced by a county is a strong predictor of its TC-attributable increases in adverse health events, potentially providing insight into strategies to minimize future TC health burdens. Our predictive models may also be useful for identifying specific communities facing the highest risk from future TC, which is critical to avert the most severe health consequences. Finally, this modeling approach can be used analogously in the context of other extreme weather and climate events, including heat waves, droughts, floods, and wildfire smoke exposures.

Methodologically, a limitation of our model is the need for user-specification of the number of latent factors, $K$. While methods exist for data-driven selection of $K$ (Bhattacharya and Dunson, 2011), they are prohibitively computationally burdensome for our application, and sensitivity analyses suggest that our results are robust to the choice of $K$. Substantively, more detailed data on the multi-dimensional TC exposures and pre-TC preparatory measures would improve the predictive ability of our models and provide greater insight into how to minimize TC health burdens. For instance, flooding is a common and often devastating TC-related exposure. While a county-level binary indicator of TC flooding is available (Anderson *and others,* 2020), this is insufficient for understanding the impact of floods, which tend to be highly localized. Additionally, mandatory prestorm evacuation orders may be a critically influential factor in the health impacts of a TC; however, to our knowledge, evacuation data have never been systematically compiled on a multi-storm scale. To minimize the health threats presented by climate and weather disasters, we must continue to collect, compile, and analyze richer data on these events.

## Supplementary Material

kxab047_Supplementary_Data

## 5. Software

R code to implement these analyses on a synthetic data set, along with complete documentation, is available at https://github.com/rachelnethery/cyclones_icpm.
