# Supplementary material for: Integrated causal-predictive machine learning models for tropical cyclone epidemiology
Source: Biostatistics. 2021 Dec 28;24(2):449–64. doi: 10.1093/biostatistics/kxab047 (PMC10102905; doi:10.1093/biostatistics/kxab047)
Supplement: kxab047_Supplementary_Data [file kxab047_supplementary_data.pdf]

S. Supplementary Information: Integrated causal-predictive machine learning models for tropical cyclone epidemiology

S.1 *Data*

S.1.1 *Health outcomes* This study focuses on the following adverse health events: all-cause mortality, respiratory hospitalizations, chronic obstructive pulmonary disorder (COPD) hospitalizations, and cardiovascular disease (CVD) hospitalizations (ICD-9/10 codes given in Table S.1). These outcomes were selected based on evidence from previous TC research and a biologically plausible link to TC exposures (Yan *and others*, 2021; Parks *and others*, 2021). Each individual's hospitalizations and death are assigned to their county of residence (not the county of hospitalization). We construct daily counts of CVD, respiratory, and COPD hospitalizations in the Medicare fee-for-service population for each county in the eastern US for the period 1999-2015. For this same set of counties, we also utilize daily county-level mortality counts in the entire Medicare population (not restricted to fee-for-service). When population denominators are used for the creation of incidence rates, the total Medicare population size is used for mortality outcomes, while the Medicare fee-for-service population size is used for hospitalization outcomes.

S.1.2 *TC exposures* To characterize county-level TC exposure, we leverage an open source data platform containing temporally-detailed track and feature data for each Atlantic-basin TC during the period 1999-2015 that came within 250km of at least one eastern US county. This data platform, which is made available through the `hurricaneexposuredata` R package, has been fully described elsewhere (Anderson *and others*, 2017, 2019, 2020). Briefly, the TC tracks are obtained from the US National Hurricane Center's "Best Tracks" dataset, which records the storm's position every 6 hours, and the storm's position is interpolated to 15-minute intervals. Wind fields are then modeled at 15-minute intervals, and the resulting values are used to estimate the storm's maximum sustained windspeed at the population centroid of each US county (see Fig-

Table S.1. ICD-9/10 codes used to define each hospitalization type.

Hospitalization Type	ICD-9	ICD-10
Cardiovascular Diseases	390.xx–398.xx, 401.xx–405.xx, 410.xx–414.xx, 415.xx–417.xx, 420.xx–429.xx	I00–I52
Respiratory Diseases	464.xx–466.xx, 480.xx–487.xx, 490.xx–492.xx, 494.xx–496.xx	J04–J06, J20–J21, J09–J18, J40–J44, J47, J67
Chronic Obstructive Pulmonary Disease	490.xx–492.xx, 494.xx–496.xx	J40, J410, J411, J449, J441, J440, J418, J42, J439, J479, J471, J670–J679

ure S.1). Cumulative precipitation amounts in each county associated with the TC are estimated by summing rainfall amounts from the North American Land Data Assimilation System Phase 2 (NLDAS-2) re-analysis dataset (Rui and Mocko, 2014) over a 4-day window beginning two days prior to the storm's closest approach to the county (these precipitation data are currently available only for years 1999-2011). The county-level wind and precipitation exposure metrics have undergone validation for use in epidemiologic studies (Anderson *and others*, 2020).

While the continuous windspeed and rainfall metrics are employed in the predictive component of the model, for the causal inference component we must define a binary metric of TC exposure, which we refer to as "treatment" for consistency with the causal inference literature. Counties exposed to the TC are referred to as treated counties and those not exposed as control counties. Because previous epidemiologic research has suggested that TC windspeed is the feature most associated with acute health impacts (Yan *and others*, 2021), we use a maximum sustained windspeed threshold to classify counties as treated or control. Moreover, because TC windfields are large and relatively homogeneous over small areas, their use to define TC exposure reduces

within-county variation and the potential for exposure misclassification. We classify a county as treated for a given storm if it experienced sustained gale-force or higher sustained wind speeds ($\geqslant$ 17.4 meters/second); otherwise, the county is classified as a control (Yan *and others*, 2021). This threshold is consistent with the outer limit wind threshold used in the US National Hurricane Center's wind radii product for characterizing tropical cyclone size.

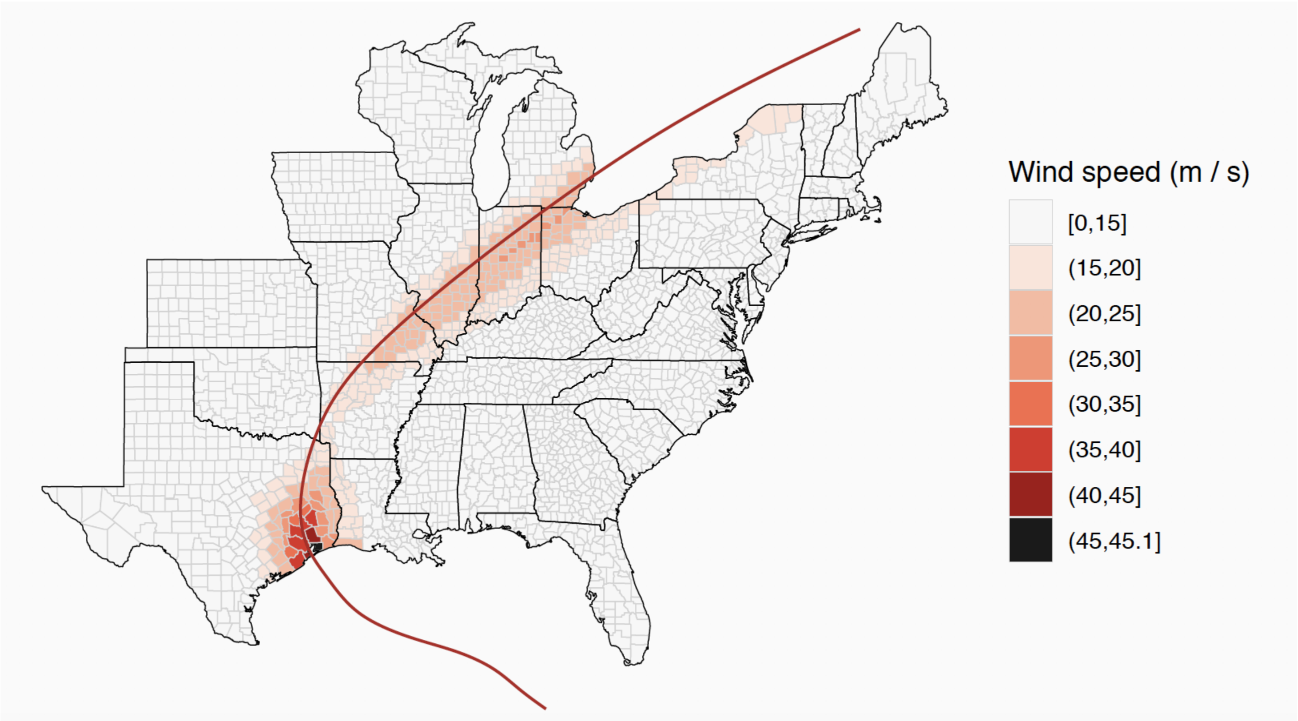

Fig. S.1. Illustration of TC track and wind exposure measure for Hurricane Ike, 2008.

S.1.3 *County and storm-specific features* Our predictive model will be used to capture the association between a TC's health impacts and the TC's meteorological features and the socioeconomic and demographic characteristics of the affected communities. To this end, for the predictive component of our model, we utilize the following TC features, which are described in more detail by Anderson *and others* (2020), for each TC-county pair (abbreviated variable

name in parentheses): modeled maximum sustained wind speed at the population centroid of the county during the TC (vmax_sust), duration of sustained wind speeds above 20 m/s at the population centroid of the county during the TC (sust_dur), year the TC occurred (year), and total number of TC exposures experienced by the county during 1999-2015 (exposure), which is a proxy for TC exposure propensity. For years 1999-2011, cumulative precipitation (precip) over a 4-day window, beginning two days prior to the TC's closest approach, is also available from the `hurricaneexposuredata` package. Because TC precipitation data are not available for our full study period, we use it only for sensitivity analyses for the predictive models.

We also consider the following spatial, demographic, and socioeconomic features of the county: percent of residents in poverty (poverty), percent of residents self-identifying as White (white_pct), percent of homes that are owner-occupied (owner_occupied), percent of residents age 65+ (age_pct_65_plus), population density (population_density), median house value (median_house_value), percent of residents without a high school degree (no_grad), a binary indicator of whether the county is located along the coastline (cc1), and the state the county is located in (state). These variables are extracted from US Census Bureau products. In particular we rely on the decennial census and American Community Survey (ACS). We align the demographic and socioeconomic features for each TC-county pair with the year of TC exposure. For years when census or ACS values are available, we use them directly, and in years not covered by the ACS or decennial census, we rely on interpolated values.

S.2 *Bayesian MC model fitting details*

Numerous Bayesian approaches to MC model fitting have been proposed (Lim and Teh, 2007; Salakhutdinov and Mnih, 2008; Gopalan *and others*, 2014; Yang *and others*, 2018), and have been studied in the context of MC for causal inference (Tanaka, 2021; Pang *and others*, 2021). Without further restrictions on the MC model, the elements of $\mathbf{U}$ and $\mathbf{V}$ are not uniquely identified (only identified up to an orthogonal rotation). The intercept terms $(\alpha, \gamma_i, \psi_t)$ are also unidentified without further constraints due to collinearity. However, in a Bayesian framework, the presence of unidentifiable parameters in the model does not compromise the estimation of identifiable parameters. Here, our interest lies in estimating $E\left[Y_{it}(0)\right]$ for missing entries, which is identifiable. Therefore, we allow these parameters to remain unidentifiable and utilize instead the posterior distribution of $E\left[Y_{it}(0)\right]$ and the posterior predictive distribution of $Y_{it}(0)$ for inference.

Bayesian estimation proceeds by first specifying a data likelihood and prior distributions for the parameters. We specify the following likelihood:

$$P(Y(0)|\mathbf{U}, \mathbf{V}, \alpha, \gamma, \psi, \eta) = \prod_i \prod_t \left[f(Y_{it}(0)|\mathbf{U}_i, \mathbf{V}_t, \alpha, \gamma_i, \psi_t, \eta)\right]^{1-D_{it}}$$

where f is a negative binomial probability mass function (pmf) with mean given by Equation 2.1, and a common scale parameter η. The form of the negative binomial pmf and its mean and variance, for a generic random variable Y, are

$$P(Y = y) = \binom{y + \eta - 1}{y} \left(\frac{\mu}{\mu + \eta}\right)^y \left(\frac{\eta}{\mu + \eta}\right)^\eta, \quad E(Y) = \mu, \quad Var(Y) = \mu + \frac{\mu^2}{\eta}.$$

We fit the MC models using rstan (Stan Development Team, 2020), and, to increase mixing and facilitate convergence, we use the default flat prior distributions in Stan. Letting U_{ki} be the element in the kth row and i^{th} column of $\mathbf{U}$ and V_{kt} be the element in the kth row and t^{th} column

of $\mathbf{V}$, then prior distributions are specified as follows:

$$
\begin{aligned}
U_{ki} &\sim Uniform(-\infty, \infty) \\
V_{kt} &\sim Uniform(-\infty, \infty) \\
\gamma_i &\sim Uniform(-\infty, \infty) \\
\psi_t &\sim Uniform(-\infty, \infty) \\
\eta &\sim Uniform(0, \infty)
\end{aligned}
\tag{S.2}
$$

For each parameter, we sample from its distribution conditional on all of the other parameters in the same causal sub-model using MCMC sampling.

S.3 *Causal identifying assumptions*

In the most general setting, the potential outcomes for our panel data would be defined as $\mathbf{Y}(\mathbf{d})$, a matrix containing all outcomes when treatment statuses for all units at all time points are given by the matrix $\mathbf{d}$. In this general case, each unit's potential outcomes depend on the treatment status of all units at all time points. In order to define the potential outcomes more concisely as we have in the paper, we invoke the classic Stable Unit Treatment Value Assumption used in causal inference (Rubin, 1980), which states that 1) there is only one version of treatment and that 2) one unit's outcome is unaffected by the treatment status of other units. If SUTVA is met, then we can define a unit's potential outcomes at time t as $Y_{it}(\mathbf{d}_i)$, where $\mathbf{d}_i$ is a binary T-length vector of the treatment status of unit i at times $1, ..., T$.

In order to define a unit's potential outcomes at time t as a function of only its treatment status at time t, as we have done in the main text, we are implicitly making the standard assumption that future treatment cannot impact past outcomes and making an additional assumption about the impact of treatment histories. That is, we assume that a unit's full historic treatment status vector is a deterministic function of the unit's time of initial treatment adoption, so a unit's potential outcomes can be expressed as functions of only the unit's time of initial treatment adoption, rather than functions of its full historic treatment status vector. The panel data construction described in the main text is designed to satisfy this assumption, by requiring that treated units initiate treatment at some time $T_0 > 1$ and remain treated through time T. In general, we can allow for varying initiation times by unit, denoted by T_{0i}. In this setting, the treatment assignment vector $\mathbf{d}_i$ is determined entirely by treatment initiation time T_{0i}, such that we can write the observed treatment vector as $\mathbf{d}_i(T_{0i})$. Under this assumption, we can write $Y_{it}(\mathbf{d}_i = \mathbf{0}) = Y_{it}(0)$, since treatment history is fully determined for a unit that has not yet initiated treatment. For treated units, because $Y_{it}(\mathbf{d}_i(T_{0i}))$ is observed when $t \geqslant T_{0i}$, it can be treated as a known quantity and simplified to $Y_{it}(1)$. Then for $i \in W,\ t \geqslant T_{0i}$ we can define the IEE as $\theta_i = \sum_{t \geqslant T_0} [Y_{it}(1) - Y_{it}(0)]$.

We posit 4 additional assumptions that are needed to identify the causal effects of a TC using the proposed matrix completion approach.

Assumption 1: Causal consistency. This assumption states that the observed outcome is equal to the potential outcome under the observed treatment level, i.e.,

$$Y_{it}(d) = Y_{it} \ \text{ if } \ D_{it} = d$$

Assumption 2: Latent ignorability. Let $\mathbf{Y}_i(\mathbf{0})$ be the vector of outcomes under control for unit i. Let $\mathbf{Z}_i$ denote observed covariates explicitly adjusted for in the causal model, if any. In our model specification, $\mathbf{Z}_i$ includes county and time indicators (corresponding to the county and time fixed effects) and the offset. Then this assumption states that conditional on $\mathbf{Z}_i$ and a generic vector of latent variables $\mathbf{R}_i$ with entries for each time $t = 1, ..., T$, the treatment assignment is independent of the potential outcomes under control. This assumption is analogous to, but weaker than, the classic ignorability assumption required for causal inference, because it allows for confounding to be captured either by observed variables, or by the time-varying latent variable. Formally, this assumption is written as

$$T_{0i} \perp\!\!\!\perp \mathbf{Y}_i(\mathbf{0}) \mid \mathbf{Z}_i, \mathbf{R}_i$$

Assumption 3: Approximation of unobservables. This assumption states that the unit-specific vectors of unobserved features $\mathbf{R}_i$ can be approximated through a low-rank latent factor model. Equivalently, this requires that the unobserved time-varying confounders can be captured by a small number of latent factors, $K << min(N, T)$. This assumption is similar to the assumption of correct model specification in standard regression models, but adapted to the context of latent variable models. Formally, this assumption requires that

$$E\left[\mathbf{Y}_i(\mathbf{0})\right] = f(\mathbf{Z}_i, \mathbf{R}_i; \pi) = f(\mathbf{Z}_i, \tilde{\mathbf{U}}_i^T \tilde{\mathbf{V}}; \pi)$$

where $\tilde{\mathbf{V}}$ is a $K \times T$ matrix whose rows contain unobserved time-varying latent factors common across units and $\tilde{\mathbf{U}}_i$ is a K-length vector of coefficients representing the influence of each latent factor on unit i. Note that we introduce the tilde notation to differentiate these "true" latent factors from those specified in our models. $f(\cdot;\pi)$ is an unspecified function of the $\mathbf{Z}_i$ and $\mathbf{R}_i$ and some parameters π. Assumption 3 would be violated if the latent $\mathbf{R}_i$ required for latent ignorability in Assumption 2 do not share a small number of common time-varying factors across units.

Assumption 4: Conditional exchangeability. Conditional on the matrices of observed covariates $\mathbf{Z} = [\mathbf{Z}_1, ..., \mathbf{Z}_N]$ and latent variables $\mathbf{R} = [\mathbf{R}_1, ..., \mathbf{R}_N]$, elements of $\mathbf{Y}(\mathbf{0})$ are exchangeable. Pang *and others* (2021) show that, under this assumption, and further conditioning on causal model parameters π, the elements of $\mathbf{Y}(\mathbf{0})$ are independent. This assumption is needed in order to define the posterior predictive distribution used to estimate the missing values in $\mathbf{Y}(\mathbf{0})$.

Under these assumptions, the causal effects are identified by Bayesian matrix completion models (Pang *and others*, 2021).

S.4 *Bayesian modularization*

Our modularized model can also be viewed as a complex Bayesian multiple imputation procedure. Causal inference in the potential outcomes framework is often treated as a missing data problem, i.e., the counterfactual outcomes are considered missing data that need to be imputed. In our case, the TC-specific causal inference models are used to impute counterfactual outcomes, and the resulting treatment effects from all the TCs are passed into the predictive model. Although the causal and predictive models suggest incompatible distributions for the missing counterfactual outcomes, the use of incompatible imputation and analysis models has been well-studied (Meng, 1994; Rubin, 2003; Schafer, 2003; Van Buuren *and others*, 2006; Van Buuren, 2007; Kuo and Wang, 2018). In the context of complex missing data, this approach, often referred to as a fully conditional model specification, incompatible Markov Chain Monte Carlo (MCMC), or multiple imputation with incongenial sources of input, has been shown to perform as well as or better than fully Bayesian data augmentation (Van Buuren *and others*, 2006; Van Buuren, 2007).

We introduce our modularization approach using this missing data perspective. Our goal is to illustrate the principles used to modularize the model by providing general forms of the modularized full conditional distributions of all parameters. We again focus on the models for a single health outcome. For a given TC s, denote by $Y_s(0)$ the full set of potential outcomes under control, composed of both the observed values $Y_s^{obs}(0)$ and the missing values $Y_s^{mis}(0)$. The causal component of the model aims to estimate the $Y_s^{mis}(0)$. Denote the TC-specific causal model covariates and parameters by ϕ_s, i.e., $\phi_s = \{i = 1, ..., N; t = 1, ...T : \mathbf{Z}_i, \mathbf{U}_i, \mathbf{V}_t, \alpha, \gamma_i, \psi_t, \eta\}_s$, and denote the treatment indicators for each unit/time collectively as D_s (note that from the missing data perspective, the treatment indicators can be viewed as missingness indicators). We assume that the $Y_s^{obs}(1)$, the (observed) potential outcomes under treatment for the treated units at post-treatment times, are fixed and known quantities so that the individual excess rates, θ_s^*, are deterministic functions of the $Y_s^{mis}(0)$. In the predictive component of the model, let X denote

the matrix of predictors and β denote the predictive model parameters. Because the $Y_s^{obs}(1)$ are considered to be fixed constants additively embedded in the predictive model outcomes θ_s^*, they can be regarded as an offset in the predictive model and absorbed into the predictor matrix X with any associated model parameters held fixed (as with a traditional offset). To illustrate in the simple case with a linear predictive model and a single post-treatment period T, where subscript siT indicates quantities corresponding to TC s, county i, and time T,

$$\theta_{si}^* = \frac{100000}{p_{iT}}(Y_{siT}^{obs}(1) - Y_{siT}^{mis}(0)) = X_i\beta \quad \Longrightarrow \quad Y_{siT}^{mis}(0) = Y_{siT}^{obs}(1) - \frac{p_{iT}}{100000}(X_i\beta)$$

So that $Y_{siT}^{obs}(1)$ can be absorbed into X_i and the associated coefficient in β held fixed. Throughout this section, to avoid confusion surrounding $Y_s^{obs}(1)$, we regard it as an offset included in X.

Then the posterior distribution of the parameters and missing data can be expressed as

$$P\left(\left\{Y_s^{mis}(0)\right\}_{s=1}^S, \left\{\phi_s\right\}_{s=1}^S, \beta \ \mid \ \left\{Y_s^{obs}(0)\right\}_{s=1}^S, \left\{D_s\right\}_{s=1}^S, X\right)$$

Traditional Bayesian MCMC sampling algorithms successively sample from the full conditional posterior distribution of each of the unknown parameters. For the missing data, the full conditional distribution is

$$\begin{aligned}
&P\left(\left\{Y_s^{mis}(0)\right\}_{s=1}^S \ \mid \ \left\{Y_s^{obs}(0)\right\}_{s=1}^S, \left\{D_s\right\}_{s=1}^S, X, \left\{\phi_s\right\}_{s=1}^S, \beta\right)\\
\propto &P\left(\left\{Y_s^{obs}(0)\right\}_{s=1}^S, \left\{Y_s^{mis}(0)\right\}_{s=1}^S, \left\{D_s\right\}_{s=1}^S, X \ \mid \ \left\{\phi_s\right\}_{s=1}^S, \beta\right)\\
= &P\left(\left\{D_s\right\}_{s=1}^S \ \mid \ \left\{Y_s^{obs}(0)\right\}_{s=1}^S, \left\{Y_s^{mis}(0)\right\}_{s=1}^S, X, \left\{\phi_s\right\}_{s=1}^S, \beta\right) \times\\
&P\left(\left\{Y_s^{obs}(0)\right\}_{s=1}^S, \ \mid \ \left\{Y_s^{mis}(0)\right\}_{s=1}^S, X, \left\{\phi_s\right\}_{s=1}^S, \beta\right) \times\\
&P\left(\left\{Y_s^{mis}(0)\right\}_{s=1}^S \ \mid \ X, \left\{\phi_s\right\}_{s=1}^S, \beta\right) \times\\
&P\left(X \ \mid \ \left\{\phi_s\right\}_{s=1}^S, \beta\right)
\end{aligned}$$

In order to modularize the model across TCs (recall that we wish to prevent all information flow between TC-specific models), we make the following assumption:

Modularization Assumption 1. We assune that the data and the missingness indicators for TC

s are independent of the data and missingness indicators for any other TC, s', given the causal model parameters for TC s, i.e.,

$$\{D_s, Y_s(0)\} \perp\!\!\!\perp \{D_{s'}, Y_{s'}(0)\}_{s' \neq s} \mid \phi_s$$

Invoking this assumption in the full conditional for $\left\{Y_s^{mis}(0)\right\}_{s=1}^S$, we obtain

$$\begin{aligned}
&P\left(\left\{Y_s^{mis}(0)\right\}_{s=1}^S \mid \left\{Y_s^{obs}(0)\right\}_{s=1}^S, \{D_s\}_{s=1}^S, X, \{\phi_s\}_{s=1}^S, \beta\right) \\
\propto &\prod_{s=1}^S \left(P\left(D_s \mid Y_s^{obs}(0), Y_s^{mis}(0), X, \phi_s, \beta\right) \times P\left(Y_s^{obs}(0) \mid Y_s^{mis}(0), X, \phi_s, \beta\right) \times P\left(Y_s^{mis}(0) \mid X, \phi_s, \beta\right)\right) \times \\
&P\left(X \mid \{\phi_s\}_{s=1}^S, \beta\right)
\end{aligned}$$

Note that $P\left(X \mid \{\phi_s\}_{s=1}^S, \beta\right)$ does not depend on $Y_s^{mis}(0)$ and thus drops out of the full conditional. By Causal Identifying Assumption 2, we have unconfoundedness conditional on the causal model parameters, so that $P\left(D_s \mid Y_s^{obs}(0), Y_s^{mis}(0), X, \phi_s, \beta\right) = P\left(D_s \mid Y_s^{obs}(0), X, \phi_s, \beta\right)$, which does not depend on $Y_s^{mis}(0)$ and also drops out. Causal Identifying Assumption 4 implies that elements of $Y_s(0)$ are independent conditional on ϕ_s, so that $Y_s^{obs}(0) \perp\!\!\!\perp Y_s^{mis}(0) \mid \phi_s$ and $P\left(Y_s^{obs}(0) \mid Y_s^{mis}(0), X, \phi_s, \beta\right) = P\left(Y_s^{obs}(0) \mid X, \phi_s, \beta\right)$, which does not contain $Y_s^{mis}(0)$ and drops out. This leaves us with the following full conditional distribution

$$\begin{aligned}
&P\left(\left\{Y_s^{mis}(0)\right\}_{s=1}^S \mid \left\{Y_s^{obs}(0)\right\}_{s=1}^S, \{D_s\}_{s=1}^S, X, \{\phi_s\}_{s=1}^S, \beta\right) \\
\propto &\prod_{s=1}^S P\left(Y_s^{mis}(0) \mid X, \phi_s, \beta\right)
\end{aligned}$$

We now make an additional modularization assumption that allows us to eliminate the predictive model parameters/predictors from this distribution.

Modularization Assumption 2. We assume that the data and missingness indicators for TC s are independent of the predictive model parameters/predictors conditional on the causal model parameters for TC s, i.e.,

$$\{D_s, Y_s(0)\} \perp\!\!\!\perp \{X, \beta\} \mid \phi_s$$

With this assumption, the full conditional distribution of the missing data can be simplified to

$$P\left(\left\{Y_s^{mis}(0)\right\}_{s=1}^S \mid \left\{Y_s^{obs}(0)\right\}_{s=1}^S, \{D_s\}_{s=1}^S, X, \{\phi_s\}_{s=1}^S, \beta\right) \propto \prod_{s=1}^S P\left(Y_s^{mis}(0) \mid \phi_s\right)$$

This distribution factorizes across TCs s and does not include the predictive model parameters, so that sampling from the posterior of $Y_s^{mis}(0)$ can proceed separately by TC and disjoint from the predictive model. In practice, because the causal model parameters, ϕ_s, are unknown, we marginalize over their posterior distribution and sample $Y_s^{mis}(0)$ from the posterior *predictive* distribution. Because both the posterior of the $Y_s^{mis}(0)$ and the posterior of the causal model parameters factorize across TCs and do not involve the predictive model parameters (shown below for the causal model parameters), the posterior predictive distribution for $Y_s^{mis}(0)$ also factorizes across TCs and does not involve the predictive model parameters.

The full conditional distribution for the $\{\phi_s\}_{s=1}^S$ is

$$\begin{aligned}
&P\left(\{\phi_s\}_{s=1}^S \mid \left\{Y_s^{obs}(0)\right\}_{s=1}^S, \left\{Y_s^{mis}(0)\right\}_{s=1}^S, \{D_s\}_{s=1}^S, X, \beta\right) \\
\propto &P\left(\left\{Y_s^{obs}(0)\right\}_{s=1}^S, \left\{Y_s^{mis}(0)\right\}_{s=1}^S, \{D_s\}_{s=1}^S, X, \{\phi_s\}_{s=1}^S, \beta\right) \\
= &P\left(\left\{Y_s^{obs}(0)\right\}_{s=1}^S, \left\{Y_s^{mis}(0)\right\}_{s=1}^S, \{D_s\}_{s=1}^S \mid X, \{\phi_s\}_{s=1}^S, \beta\right) \times \\
&P\left(X \mid \{\phi_s\}_{s=1}^S, \beta\right) \times P\left(\beta \mid \{\phi_s\}_{s=1}^S\right) \times P(\{\phi_s\}_{s=1}^S)
\end{aligned}$$

Invoking Modularization Assumption 1, we can write

$$P\left(\left\{Y_s^{obs}(0)\right\}_{s=1}^S, \left\{Y_s^{mis}(0)\right\}_{s=1}^S, \{D_s\}_{s=1}^S \mid X, \{\phi_s\}_{s=1}^S, \beta\right) = \prod_{s=1}^S P\left(Y_s^{obs}(0), Y_s^{mis}(0), D_s \mid X, \phi_s, \beta\right)$$

Then applying Causal Identifying Assumptions 2 and 4, we obtain

$$\begin{aligned}
&P\left(\left\{Y_s^{obs}(0)\right\}_{s=1}^S, \left\{Y_s^{mis}(0)\right\}_{s=1}^S, \{D_s\}_{s=1}^S \mid X, \{\phi_s\}_{s=1}^S, \beta\right) \\
= &\left(\prod_{s=1}^S P\left(Y_s^{obs}(0) \mid X, \phi_s, \beta\right) P\left(Y_s^{mis}(0) \mid X, \phi_s, \beta\right) P\left(D_s \mid X, \phi_s, \beta\right)\right)
\end{aligned}$$

Which, by Modularization Assumption 2, can be simplified to

$$P\left(\left\{Y_s^{obs}(0)\right\}_{s=1}^{S}, \left\{Y_s^{mis}(0)\right\}_{s=1}^{S}, \left\{D_s\right\}_{s=1}^{S} \mid X, \left\{\phi_s\right\}_{s=1}^{S}, \beta\right)$$
$$= \left(\prod_{s=1}^{S} P\left(Y_s^{obs}(0) \mid \phi_s\right) P\left(Y_s^{mis}(0) \mid \phi_s\right) P\left(D_s \mid \phi_s\right)\right)$$

Then plugging this term back into the full conditional equation above, and assuming independent prior distributions for the TC-specific causal parameters, we obtain

$$P\left(\left\{\phi_s\right\}_{s=1}^{S}, \mid \left\{Y_s^{obs}(0)\right\}_{s=1}^{S}, \left\{Y_s^{mis}(0)\right\}_{s=1}^{S}, \left\{D_s\right\}_{s=1}^{S}, X, \beta\right)$$
$$\propto P\left(X \mid \left\{\phi_s\right\}_{s=1}^{S}, \beta\right) \times P\left(\beta \mid \left\{\phi_s\right\}_{s=1}^{S}\right) \times$$
$$\left(\prod_{s=1}^{S} P\left(Y_s^{obs}(0) \mid \phi_s\right) P\left(Y_s^{mis}(0) \mid \phi_s\right) P\left(D_s \mid \phi_s\right) P(\phi_s)\right)$$

Following conventions in the modularization literature, we ignore the $P\left(X \mid \left\{\phi_s\right\}_{s=1}^{S}, \beta\right) \times P\left(\beta \mid \left\{\phi_s\right\}_{s=1}^{S}\right)$ term in the full conditional to prevent feedback. This modified full conditional then factorizes across TCs s and does not include the predictive model parameters, so that sampling of the ϕ_s can proceed separately by TC and disjoint from the predictive model.

Finally, the full conditional for β can be expressed as

$$P\left(\beta \mid \left\{Y_s^{obs}(0)\right\}_{s=1}^{S}, \left\{Y_s^{mis}(0)\right\}_{s=1}^{S}, \left\{D_s\right\}_{s=1}^{S}, X, \left\{\phi_s\right\}_{s=1}^{S}\right)$$
$$\propto P\left(\left\{Y_s^{obs}(0)\right\}_{s=1}^{S}, \left\{Y_s^{mis}(0)\right\}_{s=1}^{S}, \left\{D_s\right\}_{s=1}^{S}, X, \left\{\phi_s\right\}_{s=1}^{S}, \beta\right)$$
$$= P\left(\left\{Y_s^{obs}(0)\right\}_{s=1}^{S}, \left\{Y_s^{mis}(0)\right\}_{s=1}^{S} \left\{D_s\right\}_{s=1}^{S} \mid X, \left\{\phi_s\right\}_{s=1}^{S}, \beta\right) \times$$
$$P\left(X \mid \left\{\phi_s\right\}_{s=1}^{S}, \beta\right) \times P\left(\beta \mid \left\{\phi_s\right\}_{s=1}^{S}\right) \times P(\left\{\phi_s\right\}_{s=1}^{S})$$

As shown above, invoking causal identifying assumptions and modularization assumptions, we can write

$$P\left(\left\{Y_s^{obs}(0)\right\}_{s=1}^{S}, \left\{Y_s^{mis}(0)\right\}_{s=1}^{S}, \left\{D_s\right\}_{s=1}^{S} \mid X, \left\{\phi_s\right\}_{s=1}^{S}, \beta\right)$$
$$= \left(\prod_{s=1}^{S} P\left(Y_s^{obs}(0) \mid \phi_s\right) P\left(Y_s^{mis}(0) \mid \phi_s\right) P\left(D_s \mid \phi_s\right)\right)$$

Which does not contain β, so this term can be dropped. $P(\{\phi_s\}_{s=1}^{S})$ also does not depend on β and can be dropped, leaving us with

$$
\begin{aligned}
&P\left(\beta \mid \left\{Y_s^{obs}(0)\right\}_{s=1}^{S}, \left\{Y_s^{mis}(0)\right\}_{s=1}^{S}, \{D_s\}_{s=1}^{S}, X, \{\phi_s\}_{s=1}^{S}\right) \\
\propto &P\left(X \mid \{\phi_s\}_{s=1}^{S}, \beta\right) \times P\left(\beta \mid \{\phi_s\}_{s=1}^{S}\right)
\end{aligned}
$$

Thus β is sampled conditional on the causal model parameters, allowing information from the causal models to flow into the predictive model. This is the rationale that guides our modularization approach.

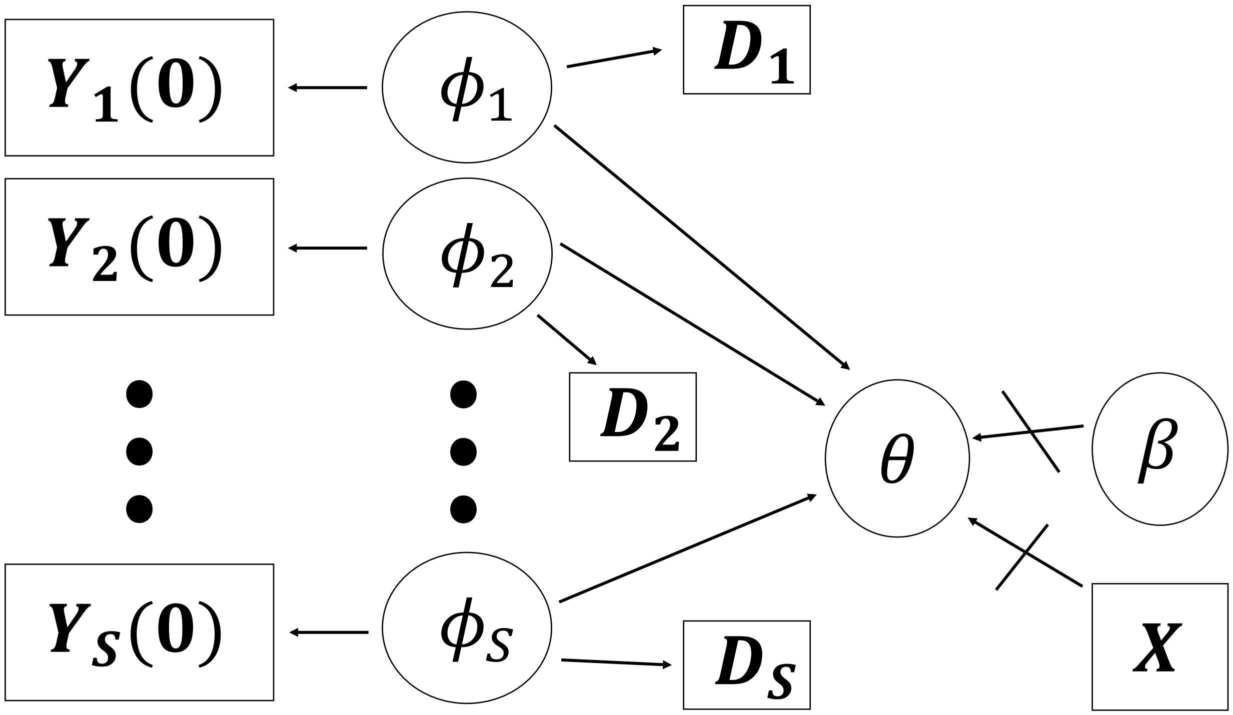

Fig. S.2. DAG for Bayesian modularization

Our full modularized model can be represented as the directed graphical model in Figure S.2. The one-way flow of information from the causal sub-models to the predictive model is depicted by the cuts in the arrows in Figure S.2. The MCMC sampling algorithm proceeds as follows (Al-

gorithm 1). First, for each TC, $s \in \{1, \dots, S\}$, we fit a separate causal sub-model (no information sharing across TCs), drawing a posterior sample of the individual excess rate for each affected county, which we denote by $\theta_{is}^{*(m)}$ for county $i \in W_s$ and posterior sample m $(m = 1, ..., M)$. We then update the predictive model parameters conditional on the individual excess rate draw, i.e., we draw a posterior sample, $\beta^{(m)}$, conditional on the $\theta_{is}^{*(m)}$ and X. We iterate this procedure until convergence of all parameters in all models.

Algorithm 1 Integrated causal-predictive model MCMC sampling algorithm

for m in $1 : M$ **do**
    for s in $1 : S$ **do**
        for i in W_s **do**
            Collect a posterior sample of the individual excess rate, $\theta_{si}^{*(m)}$, given $Y_s(0)$ and ϕ_s
        end for
    end for
    Collect a posterior sample of the predictive model parameters, $\beta^{(m)}$, given $\left\{\theta_{si}^{*(m)} : s = 1, ..., S, i \in W_s\right\}$ and X
end for

S.5 *Population displacement*

The zipcode/county of residence recorded in Medicare claims data for each recipient is updated yearly and represents their place of residence at either the end of the specified year or early the following year (timing changes slightly year-to-year). This implies that, if a person moves from county A to county B during a given year, all their hospitalizations for that year will be assigned to county B. Because the Atlantic hurricane season occurs primarily in the latter half of the year (June-November), use of the county of residence at year's end for exposure classification and population denominator construction is generally appropriate.

Yet, for a few severe storms that resulted in substantial long-term population displacement, such as Hurricane Katrina (Deryugina and Molitor, 2018), some degree of exposure misclassification is likely. However, we would expect that the impact of any such exposure misclassification would be to bias IEEs towards the null. Consider a scenario in which county A is exposed to a TC and county B is a control for that TC. Then, if a person living in county A at the time of exposure is hospitalized due to the TC, but is displaced to county B before the end of the year, their TC-attributable hospitalizations will be assigned to control county B. The resulting increase in adverse event rates in control counties and decrease in adverse event rates in treated counties should lead to an upward bias in the estimated $Y_{it}(0),\ t \geqslant T_0$, a downward bias in the observed $Y_{it}(1),\ t \geqslant T_0$, and a corresponding downward bias in the IEE for the exposed counties. In spite of this potential for underestimated IEEs, we estimate large adverse health impacts for Hurricane Katrina. For most other TCs assessed by our study, we would expect that TC-related long-term displacement rates are low, based on previous work that found that, even for unusually strong TCs (Category 3 or greater at landfall), outward migration increases on average by only 6.85% in the year following the storms (Ouattara and Strobl, 2014).

S.6 *Selection of K*

We wish to select a K value that preserves approximately 70% of the variance in the panel data matrix for all TCs and all outcomes. 70% is chosen because it represents a compromise between retaining variability necessary for accurate predictions, while not overfitting to the data. To explore the choice of K that best satisfies this criterion, we conduct exploratory principal component analyses (PCA) on the panel data matrices. Specifically, we remove the final column of the panel data matrix for each TC and outcome (because it contains missing values which are not permitted in PCA), and we implement PCA on each resulting matrix. The scree plots showing the percent of variance explained by each prinicipal component (for each panel data matrix) are shown in Figure S.3. On average across TCs, the first four principal components explain 63% of the variance in mortality, 73% in respiratory hospitalizations, 70% in COPD hospitalizations, and 74% in CVD hospitalizations. Thus $K = 4$ appears to be a reasonable choice to preserve around 70% of the variability in the panel data matrices. Moreover, as demonstrated by Figure S.3, each principal component beyond the fourth generally explains less than 10% of the variance in the panel data.

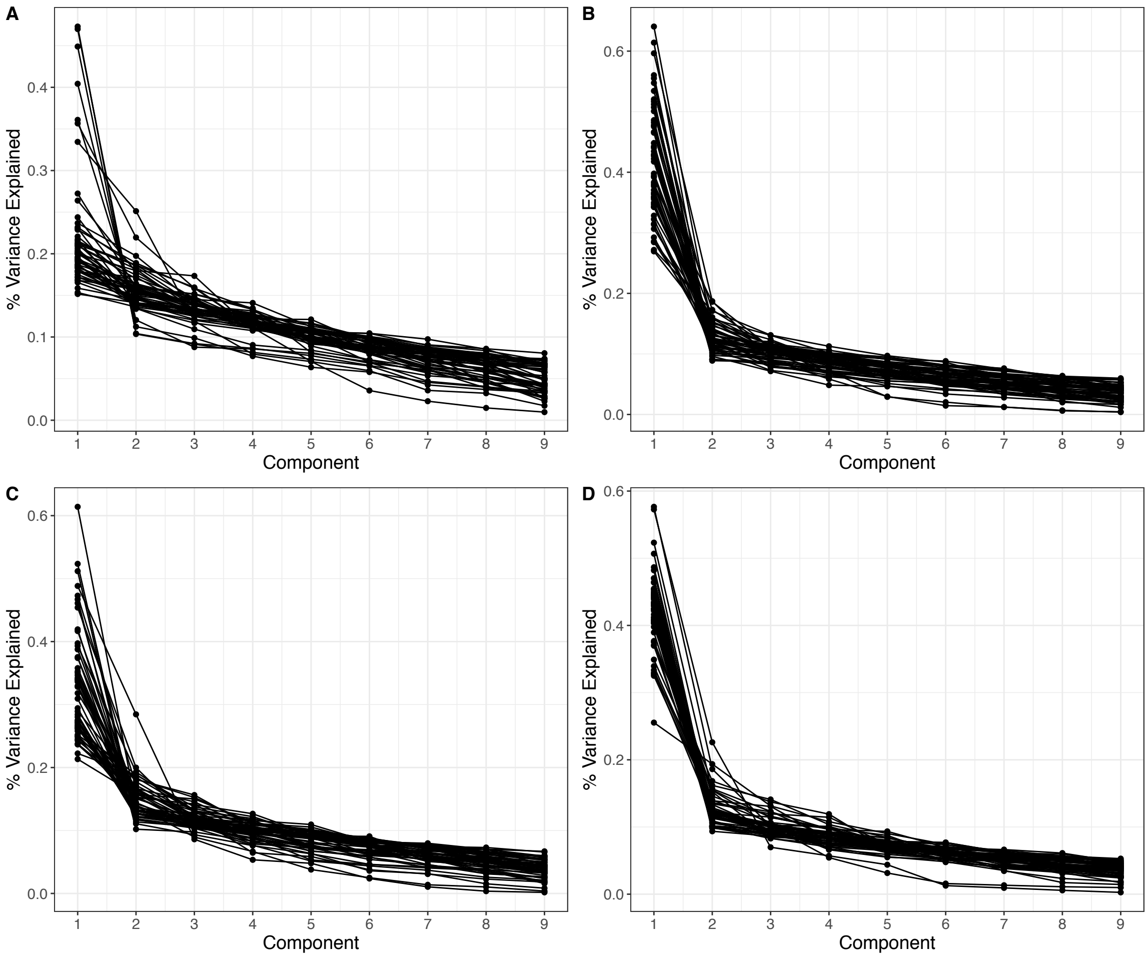

Fig. S.3. Scree plots for PCA fit to the panel data matrix for each TC for mortality (A), respiratory hospitalizations (B), COPD hospitalizations (C), and CVD hospitalizations (D).

S.7 *Predictive model selection*

Candidate predictive models are Bayesian additive regression trees (Chipman *and others*, 2010) (BART) and numerous variants of a linear regression model. In the regression models, we always include a restricted cubic spline on year to allow for flexible time trends in health effects of TCs. We consider regression models with (1) all additional predictors included as linear terms, (2) a restricted cubic spline on TC maximum sustained windspeed and all additional predictors included as linear terms, and (3) interactions between all predictors and a binary indicator of hurricane-speed winds (sustained windspeeds > 33m/s). We test each predictive model candidate both with and without state included as a predictor.

To evaluate and compare predictive models, we fit them by plugging in the county-level excess rate point estimates as the outcomes (without passing their full posterior distributions into the predictive model). Using five-fold cross-validation, we compare out-of-sample predictive performance of these models. Root mean square error for each model is given in Table S.2. For most outcomes, we find similar predictive performance across models. Overall, we find the most consistently strong performance for the regression models with splines on windspeed, excluding state. Thus, for the primary predictive sub-model, we select the Bayesian linear model with a spline on windspeed, excluding state as a predictor. For interpretability, we also provide results from a linear regression model without the windspeed spline.

Table S.2. Root mean square error from 5-fold cross-validation for each considered predictive model.

	Mortality	Resp	COPD	CVD
Linear with state	90.15	64.24	41.82	96.99
Linear without state	89.96	64.34	42.08	95.84
Spline with state	89.92	**64.15**	41.88	96.99
Spline without state	**89.59**	64.25	42.14	**95.83**
Linear, hurricane stratified	92.15	64.57	42.41	96.04
Spline, hurricane stratified	92.17	64.57	42.45	96.05
BART with state	90.85	64.28	**41.53**	95.85
BART without state	101.36	64.26	41.97	96.07

S.8 *Sensitivity analyses*

S.8.1 *Causal models* Because TC exposures are complex, our use of a binary TC exposure metric in the causal models could lead to some degree of exposure misclassification. In our models, we use as "controls" counties that are classified as unexposed and are within 150 miles of at least one exposed county. Because these controls counties lie near the path of the TC, it is likely that some of them received impacts. We anticipate that, if anything, including these counties as controls would lead to conservative results (i.e., treatment effects that are closer to the null). However, to evaluate the degree of influence this misclassification could have on our results, we fit causal models excluding from each TC's model any control county that is adjacent to an exposed county. We expect that the counties nearest to exposed counties would be most likely to experience TC impacts. Figure S.4 compares the resulting county-level excess rate estimates with the estimates from our primary models. The estimates from the two models are highly clustered around the one-to-one line for all outcomes, with no clear systematic differences. This suggests that the causal models are robust to TC exposure misclassification.

Moreover, in the causal models, results could be sensitive to the specification of the number of latent factors, K. Thus, we compare the county-level excess rate estimates obtained when specifying $K = \{3, 5\}$ to the estimates from our main model with $K = 4$. The comparison of the estimates from these models is shown in Figures S.5 and S.6. As in the previous sensitivity analysis, estimates from the models are highly clustered around the one-to-one line for all outcomes, with no clear systematic differences. This suggests that our results are robust to the specification of K.

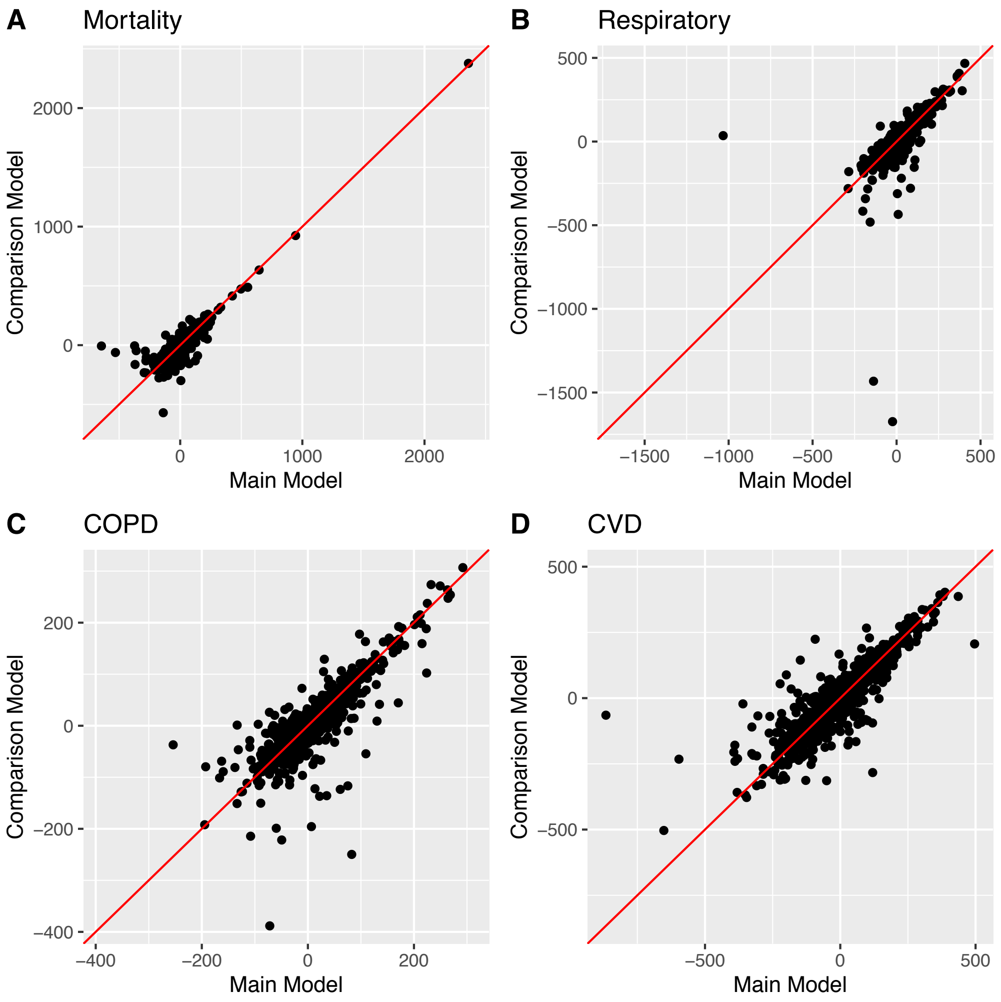

Fig. S.4. County-level excess rate estimates from the main causal models compared to causal models with counties adjacent to TC-exposed counties removed.

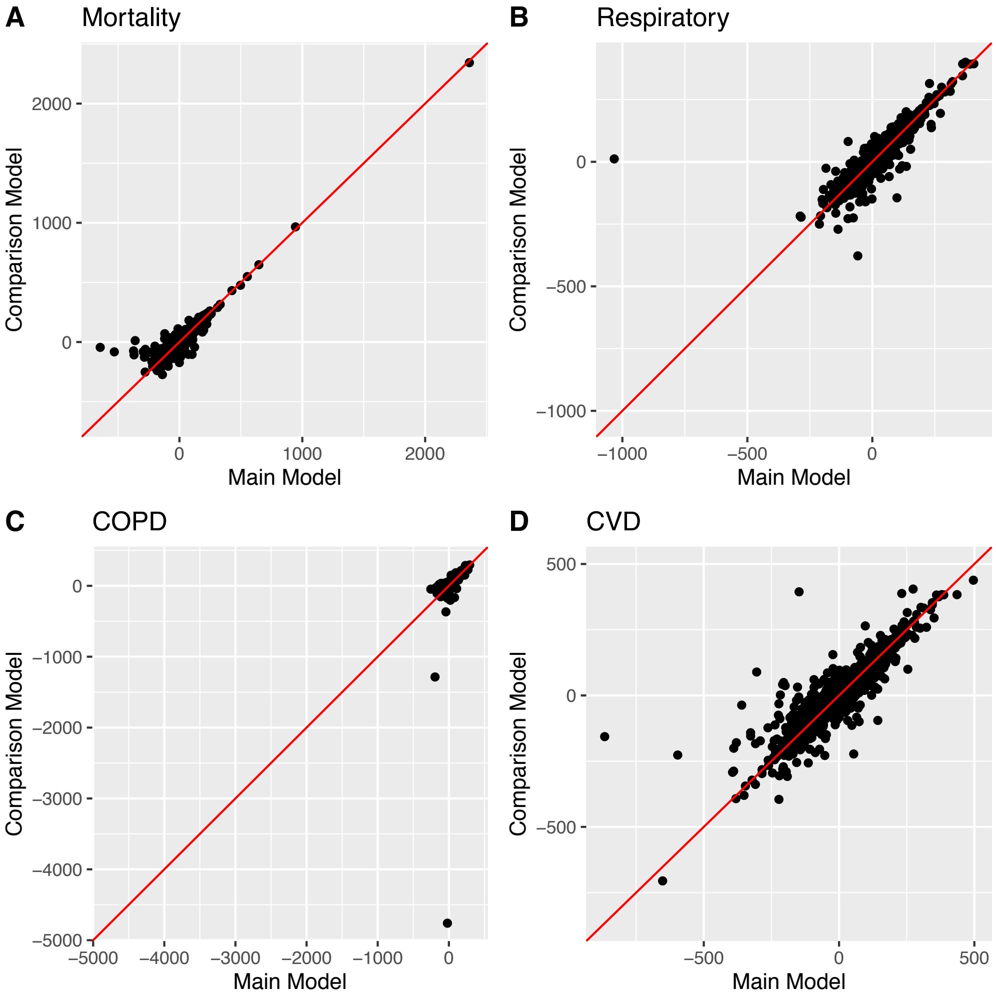

Fig. S.5. County-level excess rate estimates from the main causal models ($K = 4$) compared to causal models with $K = 3$.

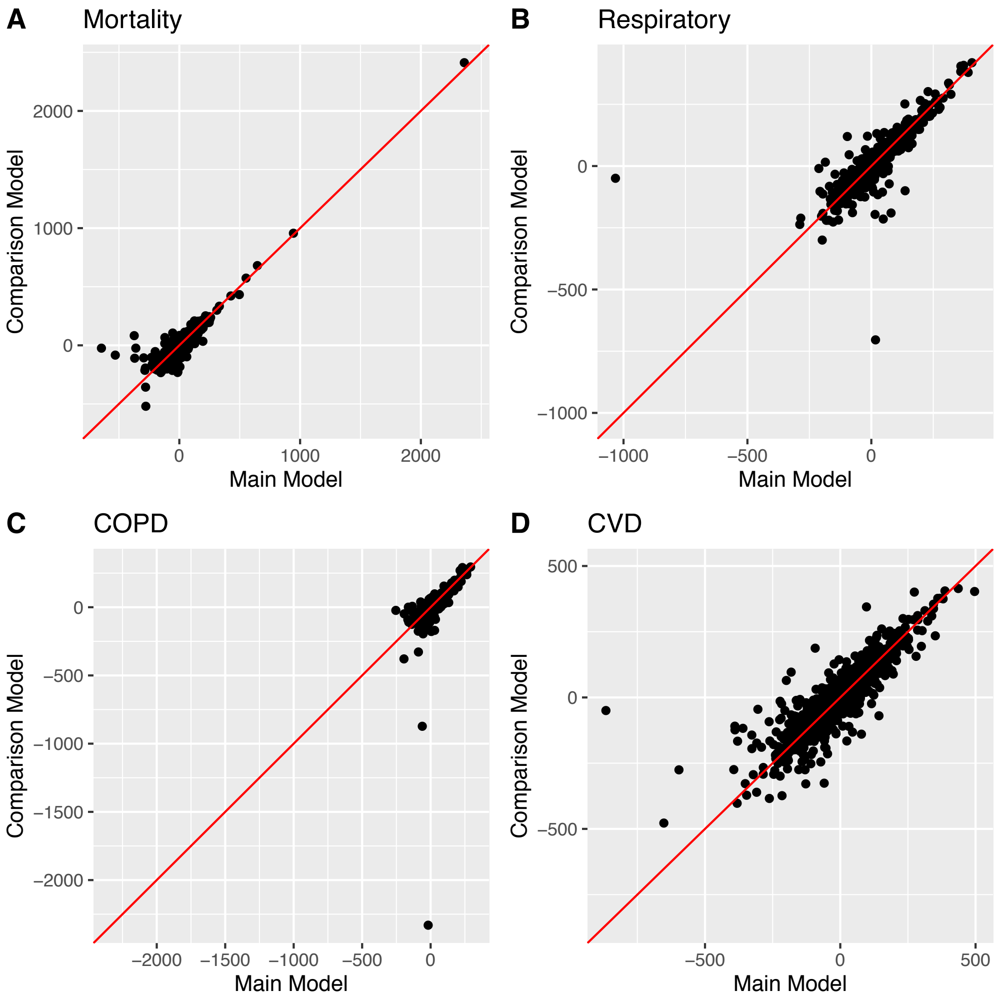

Fig. S.6. County-level excess rate estimates from the main causal models ($K = 4$) compared to causal models with $K = 5$.

S.8.2 *Predictive model* TCs can bring extreme rainfall amounts and flooding, and we hypothesize that these exposures may cause increases in some adverse health events. Currently, the `hurricaneexposuredata` package contains TC rainfall data only through the year 2011. Due to the missing precipitation data for later years, we do not include this variable in our main predictive models, but here, as an additional sensitivity analysis, we fit the predictive models only using TCs in years 2011 and prior with precipitation as a predictor. As in the primary models, we fit regression models excluding state indicators but including all other predictors, with restricted cubic splines on windspeed and year. We also include a restricted cubic spline on county-level cumulative TC precipitation, measured over a four-day window starting two days prior to the storm's closest approach to the county. The precipitation splines from the modularized models are shown in Figure S.7. After adjusting for all the other predictors, there appears to be weak or no effect of precipitation on any of the health outcomes evaluated here. Thus, the inference and predictions from our model are unlikely to be sensitive to the inclusion of precipitation information.

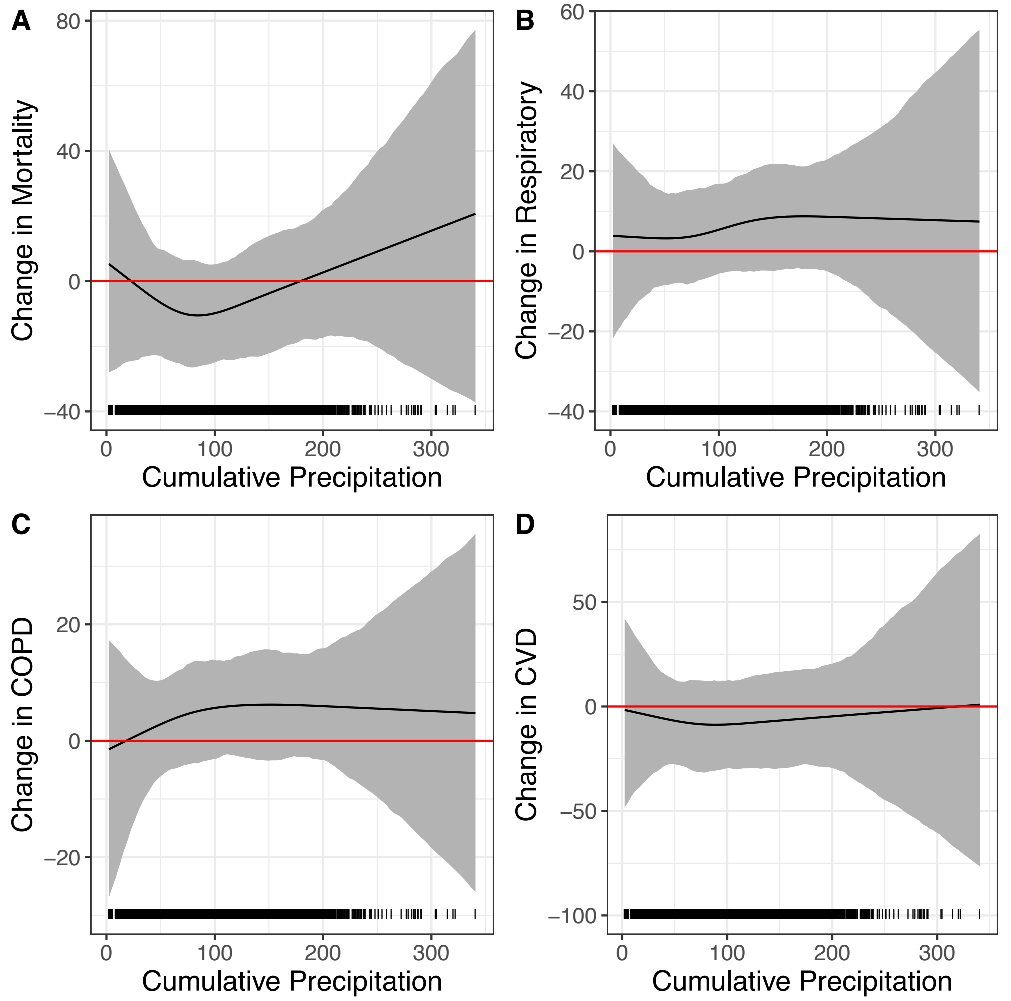

Fig. S.7. Relationship between cumulative precipitation and excess rate per 100,000 of mortality (A), respiratory hospitalizations (B), COPD hospitalizations (C), and CVD hospitalizations (D).

S.9 *Additional tables and figures*

Table S.3. Definition of estimands. $s = \{1, ..., S\}$ indexes TCs and $i \in W_s$ indexes treated counties for TC s.

Name	Definition	Formula
Individual excess events (IEE)	County-level excess events attributable to a single TC s over the treatment period	$\theta_{si} = \sum_{t \geqslant T_0} [Y_{sit}(1) - Y_{sit}(0)]$
Individual excess rate	County-level excess event rate (per 100,000 population) attributable to a single TC s over the treatment period	$\theta^*_{si} = 100000 \times (\theta_{si}/p_{iT})$
TC-specific excess events	Cumulative excess events summed across all counties impacted by a single TC s	$\sum_{i \in W_s} \theta_{si}$
TC-specific excess rate	Excess event rate (per 100,000 population) across all counties impacted by a single TC s	$100000 \times (\sum_{i \in W_s} p_{iT})^{-1} \sum_{i \in W_s} \theta_{si}$
Total excess events (TEE)	Cumulative TC-attributable excess events summed over all TCs and counties in the study	$TEE = \sum_{s=1}^{S} \sum_{i \in W_s} \theta_{si}$
Average excess rate (AER)	Average of the TC-attributable excess rates across all county-level TC exposures in the study	$AER = \frac{1}{N_{total}} \sum_{s=1}^{S} \sum_{i \in W_s} \theta^*_{si}$, $N_{total} = \sum_{s=1}^{S} \lvert W_s \rvert$ is the total number of county-level TC exposures in our analyses

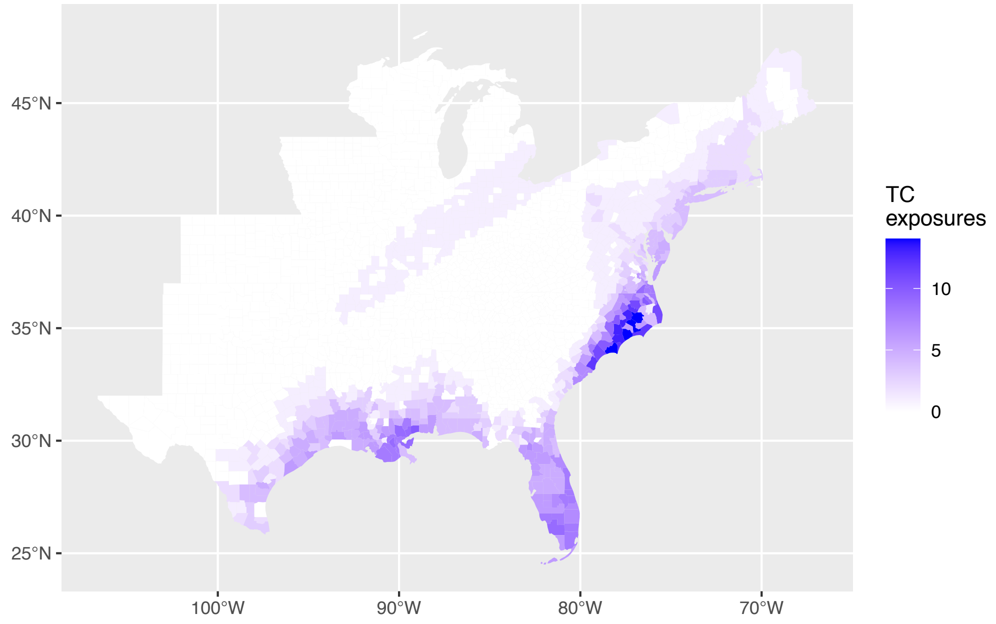

Fig. S.8. Number of Atlantic Basin TC exposures included in our analyses (by county), 1999-2015.

Table S.4. Name and year of each TC included in the study, the number of treated and control counties for the TC, and the rates (per 100,000) of each health outcome in Medicare in the treated and control counties during the 140 day period surrounding the TC.

TC	N Trt	N Ctl	Mort Trt	Mort Ctl	Resp Trt	Resp Ctl	COPD Trt	COPD Ctl	CVD Trt	CVD Ctl
Alberto-2006	21	130	2036	1901	1361	1088	494	413	3210	2779
Alex-2004	18	86	1828	1858	1052	909	380	331	2882	2868
Allison-2001	22	143	2161	2209	1483	2050	584	743	3433	3832
Ana-2015	3	52	1476	1751	800	875	369	355	1838	1971
Andrea-2013	80	223	1718	1667	899	850	373	356	2059	1897
Arlene-2005	4	61	1871	2189	1472	1805	561	669	3010	3288
Arthur-2014	45	113	1642	1541	618	607	248	251	1864	1579
Barry-2001	18	59	1971	2099	1412	1397	556	554	3418	3552
Barry-2007	8	73	1778	1841	729	947	272	370	2187	2616
Beryl-2012	22	114	1638	1664	853	897	366	409	1996	2118
Bill-2003	16	77	2139	2056	1400	1581	546	554	3786	3495
Bill-2015	9	47	1793	1585	929	852	343	322	1877	1708
Bret-1999	15	28	1611	1903	1015	995	386	362	3101	2863
Charley-2004	76	120	1758	1801	849	918	341	333	2980	2804
Cindy-2005	13	78	2330	2004	1357	1562	489	560	3247	3086
Claudette-2003	36	41	1914	1866	1163	1433	399	531	3018	3370
Claudette-2009	3	53	1700	1787	1056	996	489	464	2713	2535
Dennis-1999	23	94	1792	1864	988	904	427	356	3058	2859
Dennis-2005	35	81	1990	2105	1452	1360	541	506	3254	2858
Dolly-2008	13	22	1617	1659	1123	783	429	290	2536	2088
Edouard-2008	10	72	2025	1767	1216	966	502	400	2565	2435
Ernesto-2006	72	192	1698	1734	787	789	307	296	2494	2653
Fay-2002	2	47	2047	1853	796	995	189	381	3541	3010
Fay-2008	52	50	1634	1794	763	884	354	409	2321	2370
Floyd-1999	142	156	1765	1824	904	890	315	325	2776	2913
Frances-2004	50	39	1734	1843	842	974	352	391	2960	3178
Gabrielle-2001	23	26	1802	1827	851	930	349	388	3073	3256
Gabrielle-2007	6	71	1696	1770	763	715	360	273	2292	2413
Gaston-2004	25	116	1761	1788	879	819	336	281	2855	2701
Gordon-2000	12	63	1862	1775	817	826	371	343	3018	3111
Gustav-2008	48	77	1844	1761	1062	914	431	392	2811	2341
Hanna-2002	2	74	1871	1973	960	1198	388	478	3397	3339
Hanna-2008	127	163	1660	1681	763	759	325	321	2470	2202
Harvey-1999	3	20	1876	1739	1309	823	535	334	3300	2950
Helene-2000	14	96	1988	1913	1089	960	418	381	3139	2983
Hermine-2010	13	30	1515	1599	891	742	369	295	2063	1897
Humberto-2007	31	81	1766	1838	942	956	334	329	2490	2580
Ike-2008	215	64	1782	1756	924	874	411	350	2471	2414
Irene-1999	28	125	1777	1820	1015	875	411	360	3119	2889
Irene-2011	146	146	1622	1623	793	755	329	313	2046	1884
Isaac-2012	41	70	1769	1785	888	936	343	393	2068	2062
Isabel-2003	135	86	1857	1890	1008	1036	362	378	2778	3079
Isidore-2002	25	80	2025	1981	1200	1221	486	463	3627	3291
Ivan-2004	48	72	1867	1959	1108	989	472	376	3428	3018
Jeanne-2004	45	35	1731	1830	834	874	352	346	2977	3020
Katrina-2005	78	72	1875	1781	957	902	370	338	2726	2856
Lee-2011	30	91	1769	1785	841	969	372	406	2159	2136
Lili-2002	34	86	1982	1962	1248	1148	452	447	3565	3228
Ophelia-2005	23	112	1687	1728	872	819	344	307	2782	2629
Rita-2005	40	89	1803	1823	989	866	381	322	2760	2687
Sandy-2012	105	121	1606	1671	683	718	288	297	1911	1843
Tammy-2005	12	52	1789	1734	873	793	313	315	2847	2697
Wilma-2005	18	21	1689	1735	770	767	305	310	2603	2714

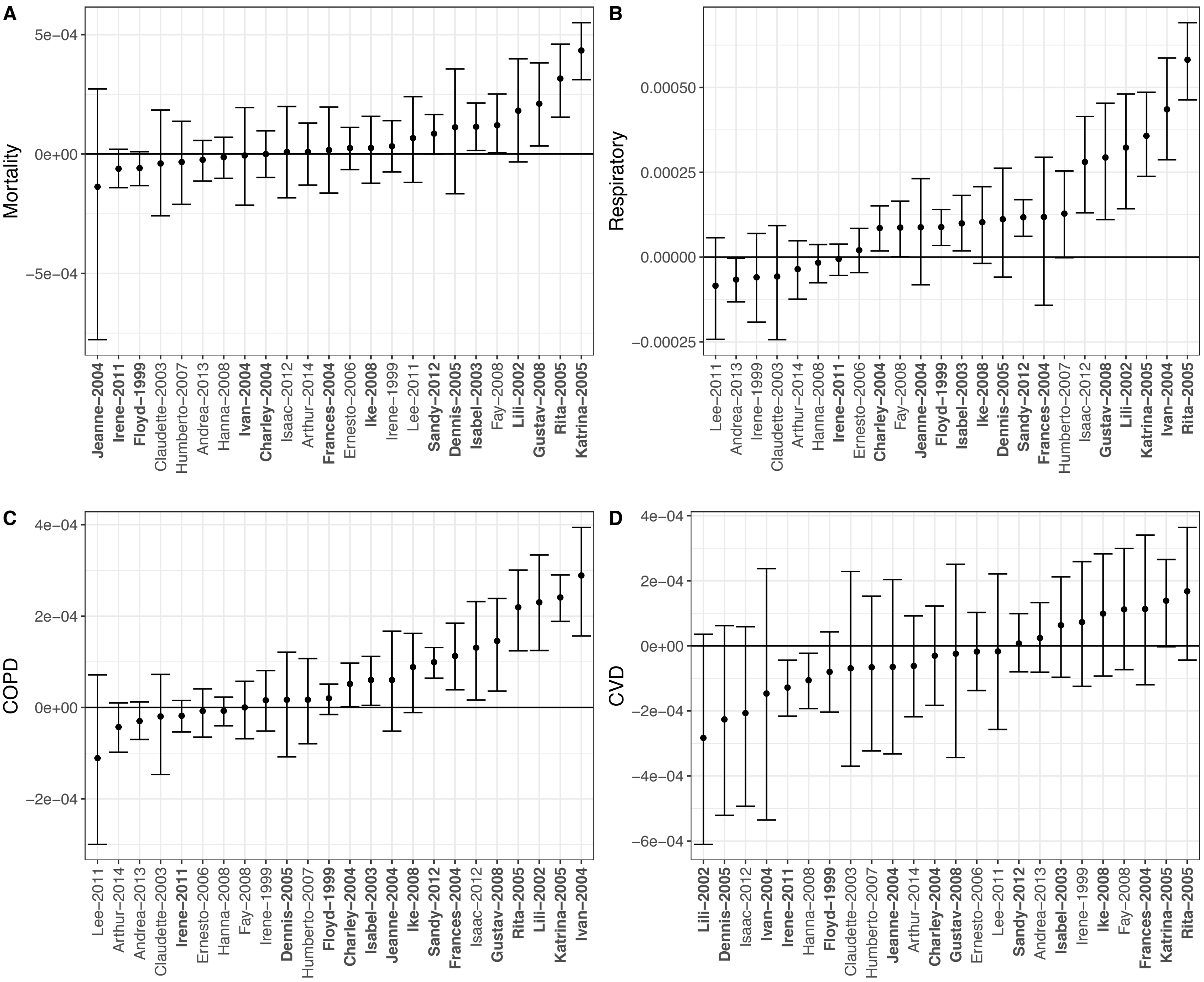

Fig. S.9. TC-specific excess rate estimates and 95% predictive intervals for mortality (A), respiratory hospitalizations (B), COPD hospitalizations (C), and CVD hospitalizations (D) for TCs that impacted > 25 counties. The TC-specific excess rate is the rate of excess events across the total population impacted by the TC. Bolded TC labels indicate storm names that were subsequently retired– retirement occurs when a TC is so destructive that re-using the name is considered to be insensitive (National Hurricane Center, 2020).

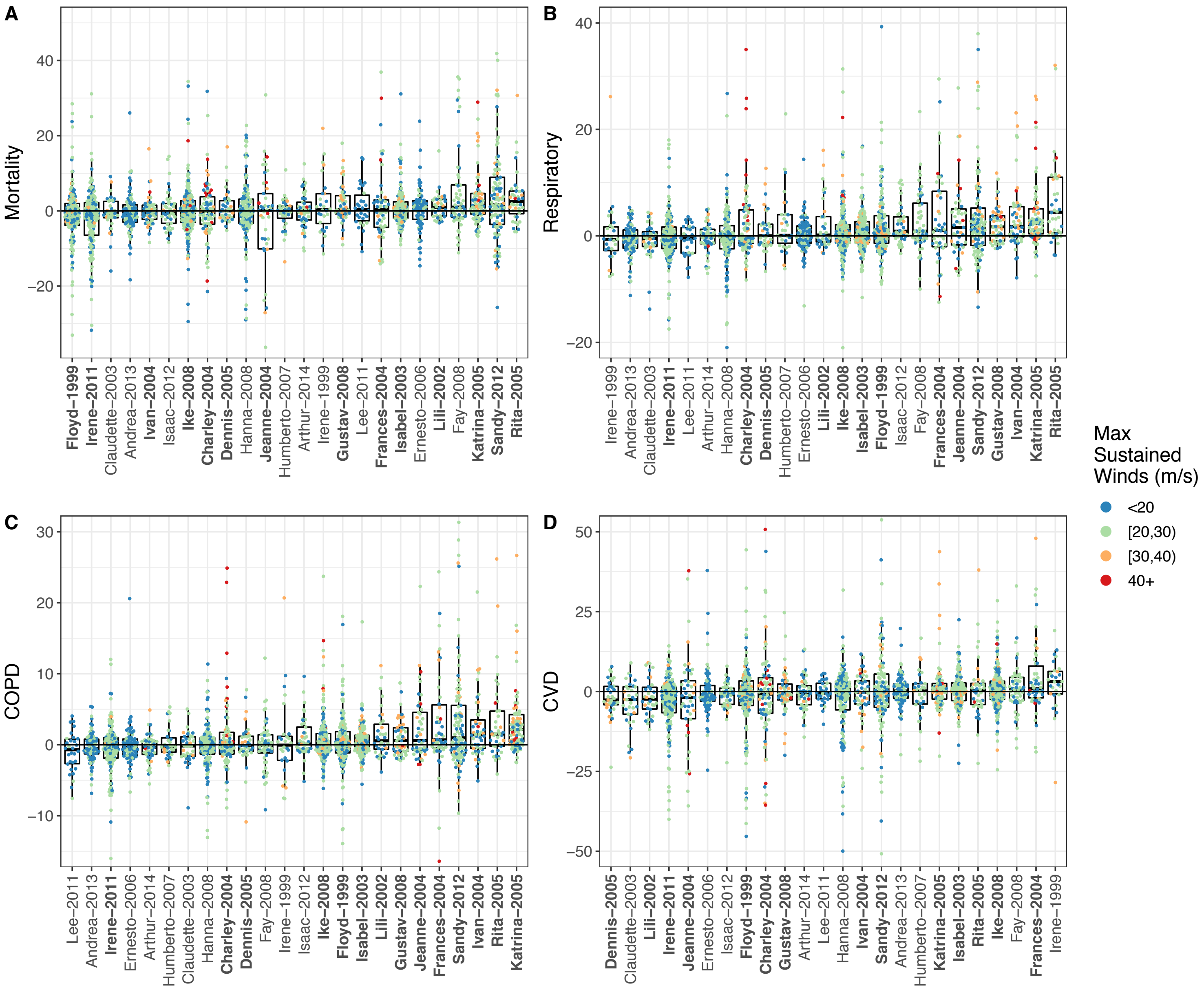

Fig. S.10. County-level individual excess events (IEE) estimates for mortality (A), respiratory hospitalizations (B), COPD hospitalizations (C), and CVD hospitalizations (D) for TCs that impacted > 25 counties. The IEE is the estimated number of excess events in the county due to the TC. Distant outliers are cropped out for readability. Bolded TC labels indicate storm names that were subsequently retired–retirement occurs when a TC is so destructive that re-using the name is considered to be insensitive (National Hurricane Center, 2020).

Table S.5. Predictive model coefficient posterior means (95% CIs) from models with a natural cubic spline on sustained windspeed and year, not adjusted for state. Variable names with suffix _s are components of a restricted cubic spline basis for the variable.

	Mortality	Resp	COPD	CVD
(Intercept)	-115.89 (-378.81, 107.09)	39.13 (-113.1, 198.02)	18.07 (-117.22, 137.78)	-45.97 (-333.02, 243.32)
vmax_sust_s1	4.34 (-7.09, 17.48)	-0.97 (-8.93, 6.6)	0.43 (-5.6, 6.75)	2.38 (-11.82, 15.2)
vmax_sust_s2	-92.36 (-248.23, 37.85)	-6.37 (-97.13, 87.88)	7.56 (-69.23, 80.78)	-41.31 (-194, 129.42)
vmax_sust_s3	181.19 (-46.76, 449.3)	19.6 (-143.67, 180.82)	-12.29 (-140.88, 124.59)	78.09 (-221.95, 345.87)
poverty	35.26 (-149.01, 206.88)	114.78 (-22.65, 244.98)	20.54 (-79.69, 119.58)	-121.81 (-375.06, 110.56)
white_pct	14.23 (-38.78, 64.42)	-24.13 (-62.07, 12.89)	-32.12 (-61.3, -4.57)	17.56 (-51.28, 89.96)
owner_occupied	-16.78 (-115.76, 84.09)	17.94 (-60.87, 89.38)	38.48 (-20.04, 95.68)	-91.02 (-239.35, 49.81)
age_pct_65_plus	-165.94 (-486.81, 139.45)	7.66 (-241.24, 225.26)	36.44 (-141.45, 213.65)	-95.8 (-514.39, 300.21)
median_age	1.26 (-2.29, 5.05)	0.02 (-2.27, 2.52)	-0.49 (-2.4, 1.57)	1.72 (-2.57, 6.62)
population_density	-0.58 (-7.57, 6.61)	0.02 (-5.34, 4.98)	0.82 (-3.13, 4.8)	-0.97 (-9.88, 7.72)
median_house_value	2.83 (-7.67, 12.33)	0.62 (-5.89, 7.82)	-0.38 (-6.05, 5.11)	-5.61 (-18.46, 7.29)
no_grad	14.72 (-137.05, 154.17)	-62.79 (-167.04, 40.83)	-25.48 (-106.23, 58.74)	36.94 (-143.44, 207.96)
year_s1	-1.78 (-17.02, 14.35)	6.97 (-4.91, 19.73)	6.64 (-2.85, 15.56)	-2.08 (-23.65, 17.19)
year_s2	-2.92 (-21.43, 13.73)	-11.03 (-24.57, 1.7)	-8.55 (-18.28, 1.86)	4.73 (-16.43, 27.76)
exposure	1.19 (-0.78, 3.25)	-1.61 (-3.02, -0.17)	-1.29 (-2.35, -0.24)	0.17 (-2.12, 2.84)
sust_dur	0.01 (-0.02, 0.05)	0.03 (0.01, 0.06)	0 (-0.02, 0.02)	-0.01 (-0.05, 0.04)
cc1	-2.37 (-16.09, 12.75)	-1.24 (-11.75, 9.38)	-2.17 (-9.82, 5.43)	8.19 (-9.89, 27.44)

Table S.6. Predictive model coefficient posterior means (95% CIs) from models linear models (spline term only on the year variable), not adjusted for state. Variable names with suffix _s are components of a restricted cubic spline basis for the variable.

	Morality	Resp	COPD	CVD
(Intercept)	-101.38 (-216.44, 8.75)	-6.18 (-84.45, 74.71)	0.89 (-57.79, 60.08)	-19.05 (-155.97, 118.74)
vmax_sust	3.21 (1.59, 5.09)	1.39 (0.16, 2.59)	1.36 (0.42, 2.28)	0.84 (-1.24, 3.08)
sust_dur	-0.01 (-0.05, 0.02)	0.02 (0, 0.04)	0 (-0.01, 0.02)	-0.01 (-0.05, 0.03)
exposure	1.36 (-0.64, 3.3)	-1.59 (-2.96, -0.27)	-1.28 (-2.37, -0.2)	0.27 (-2.08, 2.76)
poverty	48.39 (-135.71, 228.03)	118.2 (-15.32, 241.09)	22.36 (-82.63, 125.13)	-116.76 (-366.75, 110.8)
white_pct	12.55 (-39.29, 64.74)	-25.91 (-66.17, 10.07)	-32.12 (-61.68, -3.08)	17.14 (-50.02, 88.44)
owner_occupied	-4.82 (-110.78, 98.17)	21.51 (-50.72, 97.63)	38.45 (-16.56, 96.45)	-90.14 (-233.67, 52.13)
age_pct_65_plus	-123.03 (-446.07, 210.72)	27.06 (-207.2, 253.55)	44.02 (-133.55, 217.4)	-91.54 (-519.72, 298.64)
median_age	0.78 (-2.99, 4.59)	-0.19 (-2.63, 2.31)	-0.53 (-2.54, 1.49)	1.68 (-2.75, 6.38)
population_density	-1.1 (-7.94, 5.71)	-0.36 (-5.12, 5.01)	0.75 (-2.95, 4.46)	-1.22 (-10.09, 7.51)
median_house_value	3.64 (-6.72, 13.2)	0.84 (-6.38, 7.94)	-0.19 (-5.42, 5.52)	-5.52 (-18.1, 7.11)
no_grad	19.87 (-126.09, 162.17)	-57.28 (-163.61, 47.03)	-26.03 (-108.24, 52.87)	37.35 (-136.49, 216.22)
year_s1	-0.85 (-17.39, 15.05)	7.34 (-3.76, 18.99)	6.72 (-3.06, 15.65)	-1.08 (-21.94, 19.25)
year_s2	-3.84 (-21.69, 15.86)	-11.46 (-24.17, 1.48)	-8.8 (-18.86, 1.73)	3.62 (-20.64, 27.44)
cc1	-1.23 (-16.32, 13.4)	-1.05 (-11.84, 9.45)	-2.47 (-10.36, 5.16)	8.5 (-9.28, 27)

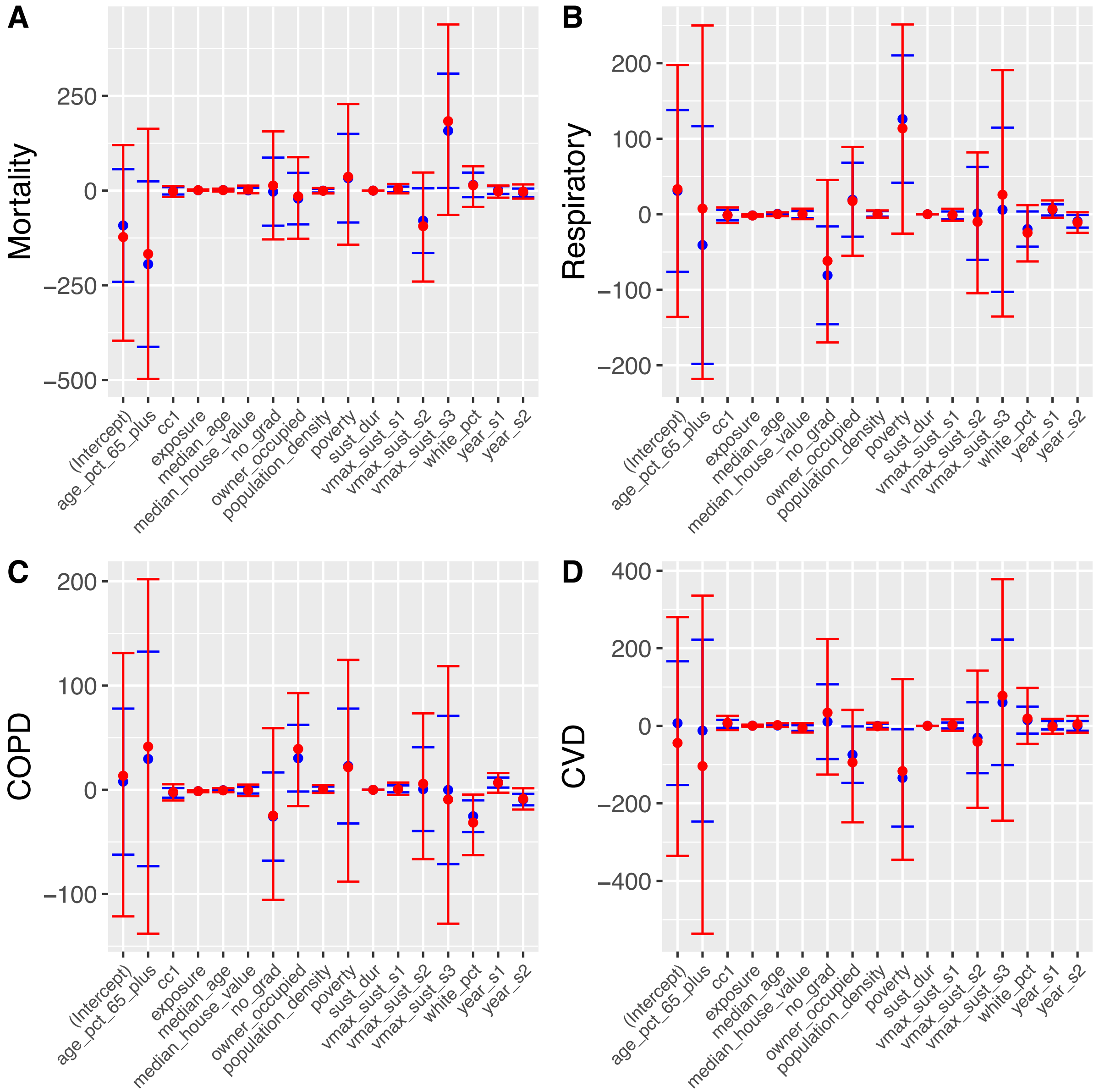

Fig. S.11. Point estimates and 95% CIs from predictive models for each outcome that do propagate uncertainty from the causal models (red) and do not propagate uncertainty (blue).

Supplemental references

Anderson, Brooke, Schumacher, Andrea, Crosson, William, Al-Hamdan, Mohammad, Yan, Meilin, Ferreri, Joshua, Chen, Ziyu, Quiring, Steven and Guikema, Seth. (2017). *hurricaneexposuredata: Data Characterizing Exposure to Hurricanes in United States Counties*. R package version 0.0.2.

Anderson, Brooke, Yan, Meilin, Ferreri, Joshua, Crosson, William, Al-Hamdan, Mohammad, Schumacher, Andrea and Eddelbuettel, Dirk. (2019). *hurricaneexposure: Explore and Map County-Level Hurricane Exposure in the United States*. R package version 0.1.0.

Anderson, G Brooke, Ferreri, Joshua, Al-Hamdan, Mohammad, Crosson, William, Schumacher, Andrea, Guikema, Seth, Quiring, Steven, Eddelbuettel, Dirk, Yan, Meilin and Peng, Roger D. (2020). Assessing United States county-level exposure for research on tropical cyclones and human health. *Environmental Health Perspectives* **128**(10), 107009.

Chipman, Hugh A, George, Edward I, McCulloch, Robert E *and others*. (2010). Bart: Bayesian additive regression trees. *The Annals of Applied Statistics* **4**(1), 266–298.

Deryugina, Tatyana and Molitor, David. (2018). Does when you die depend on where you live? evidence from hurricane katrina. *Technical Report*, National Bureau of Economic Research.

Gopalan, Prem K, Charlin, Laurent and Blei, David. (2014). Content-based recommendations with Poisson factorization. In: *Advances in Neural Information Processing Systems*. pp. 3176–3184.

Kuo, Kun-Lin and Wang, Yuchung J. (2018). Simulating conditionally specified models. *Journal of Multivariate Analysis* **167**, 171–180.

Lim, Yew Jin and Teh, Yee Whye. (2007). Variational Bayesian approach to movie rating prediction. In: *Proceedings of KDD cup and workshop*, Volume 7. Citeseer. pp. 15–21.

Meng, Xiao-Li. (1994). Multiple-imputation inferences with uncongenial sources of input. *Statistical Science*, 538–558.

National Hurricane Center. (2020 (accessed October 14, 2020)). *Tropical Cyclone Naming History and Retired Names*.

Ouattara, Bazoumana and Strobl, Eric. (2014). Hurricane strikes and local migration in us coastal counties. *Economics Letters* **124**(1), 17–20.

Pang, Xun, Liu, Licheng and Xu, Yiqing. (2021). A Bayesian alternative to synthetic control for comparative case studies. *Political Analysis*, 1–20.

Parks, Robbie M, Anderson, G Brooke, Nethery, Rachel C, Navas-Acien, Ana, Dominici, Francesca and Kioumourtzoglou, Marianthi-Anna. (2021). Tropical cyclone exposure is associated with increased hospitalization rates in older adults. *Nature Communications* **12**(1), 1–12.

Rubin, Donald B. (1980). Randomization analysis of experimental data: The fisher randomization test comment. *Journal of the American Statistical Association* **75**(371), 591–593.

Rubin, Donald B. (2003). Nested multiple imputation of nmes via partially incompatible mcmc. *Statistica Neerlandica* **57**(1), 3–18.

Rui, H and Mocko, D. (2014). Readme Document for North America Land Data Assimilation System Phase 2 (NLDAS-2) Products. *Greenbelt, Maryland*.

Salakhutdinov, Ruslan and Mnih, Andriy. (2008). Bayesian probabilistic matrix factorization using Markov chain Monte Carlo. In: *Proceedings of the 25th international conference on Machine learning*. pp. 880–887.

Schafer, Joseph L. (2003). Multiple imputation in multivariate problems when the imputation and analysis models differ. *Statistica Neerlandica* **57**(1), 19–35.

Stan Development Team. (2020). RStan: the R interface to Stan. R package version 2.19.3.

Tanaka, Masahiro. (2021). Bayesian matrix completion approach to causal inference with panel data. *Journal of Statistical Theory and Practice* **15**(2), 1–22.

Van Buuren, Stef. (2007). Multiple imputation of discrete and continuous data by fully conditional specification. *Statistical methods in medical research* **16**(3), 219–242.

Van Buuren, Stef, Brand, Jaap PL, Groothuis-Oudshoorn, Catharina GM and Rubin, Donald B. (2006). Fully conditional specification in multivariate imputation. *Journal of statistical computation and simulation* **76**(12), 1049–1064.

Yan, Meilin, Wilson, Ander, Dominici, Francesca, Wang, Yun, Al-Hamdan, Mohammad, Crosson, William, Schumacher, Andrea, Guikema, Seth, Magzamen, Sheryl, Peel, Jennifer L *and others*. (2021). Tropical cyclone exposures and risks of emergency Medicare hospital admission for cardiorespiratory diseases in 175 urban United States counties, 1999–2010. *Epidemiology* **32**(3), 315–326.

Yang, Linxiao, Fang, Jun, Duan, Huiping, Li, Hongbin and Zeng, Bing. (2018). Fast low-rank Bayesian matrix completion with hierarchical Gaussian prior models. *IEEE Transactions on Signal Processing* **66**(11), 2804–2817.

[*Received May XX, 2021*]
